# Supplementary figures and images for: Genome-wide identification and comprehensive analysis of the phytochrome-interacting factor (PIF) gene family in wheat
Source: PLoS One. 2024 Jan 5;19(1):e0296269. doi: 10.1371/journal.pone.0296269 (PMC10769075; doi:10.1371/journal.pone.0296269)

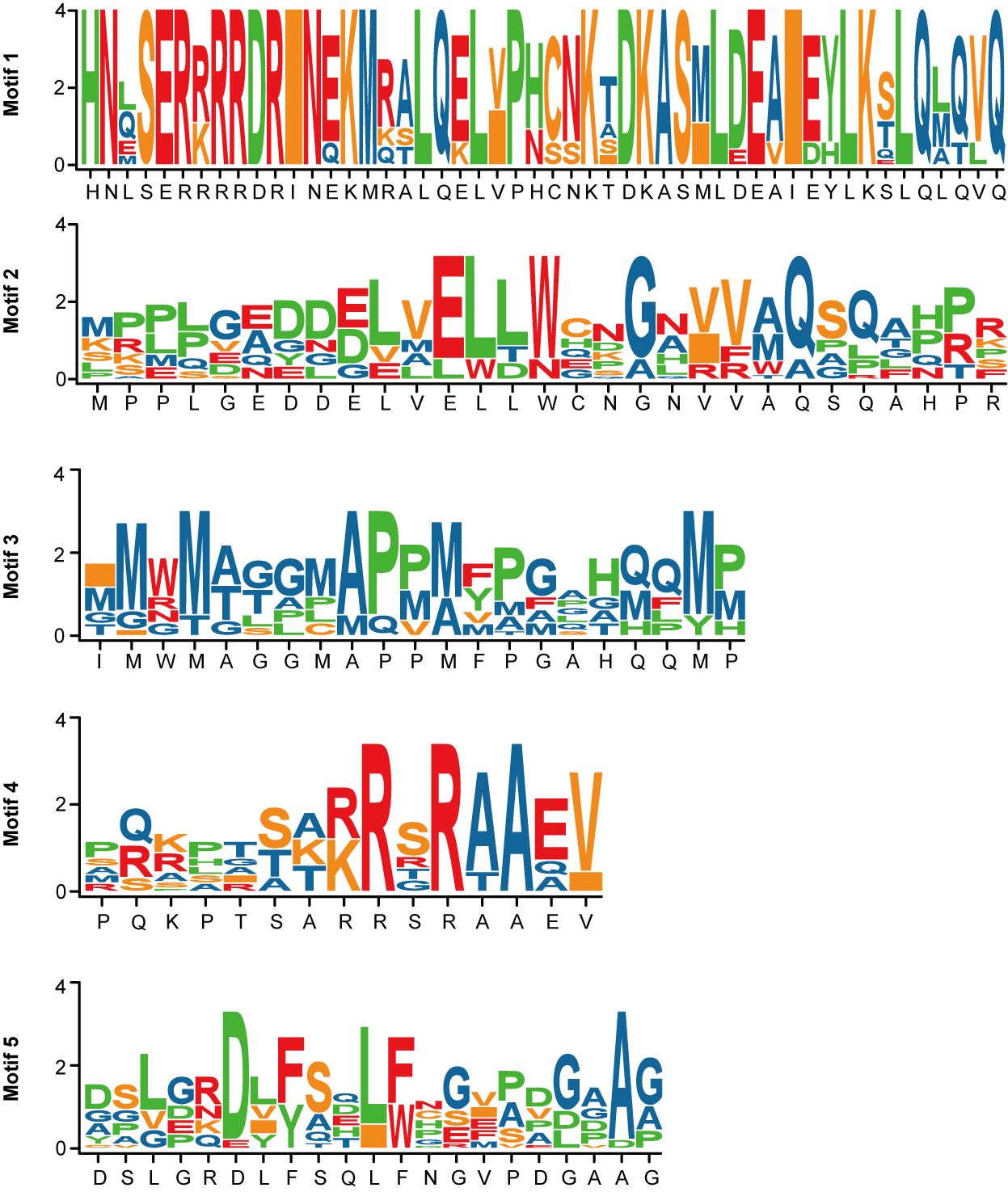

Supplement: S2 Fig — (TIF) [file pone.0296269.s002.tif]
