# Supplementary material for: Genome-wide identification and comprehensive analysis of the phytochrome-interacting factor (PIF) gene family in wheat
Source: PLoS One. 2024 Jan 5;19(1):e0296269. doi: 10.1371/journal.pone.0296269 (PMC10769075; doi:10.1371/journal.pone.0296269)
Supplement: Attachment — Submitted filename: PONE-D-23-27525.pdf [file pone.0296269.s010.pdf]

PLOS ONE

Genome-wide identification and comprehensive analysis of the phytochrome-interacting factor (PIF) gene family in wheat

--Manuscript Draft--

**Manuscript Number:**	PONE-D-23-27525
**Article Type:**	Research Article
**Full Title:**	Genome-wide identification and comprehensive analysis of the phytochrome-interacting factor (PIF) gene family in wheat
**Short Title:**	The phytochrome-interacting factor (PIF) gene family in wheat
**Corresponding Author:**	Qing tian Chen, ph.D<br>Shaanxi Provincial Land Engineering Construction Group<br>Xi'an, CHINA
**Keywords:**	Wheat; PIFs; phytochrome-interacting factors; expression analysis; abiotic stress
**Abstract:**	Phytochrome-interacting factors (PIFs) are essential transcription factors for plant growth, development, and stress responses. Although PIF genes have been well-studied in many plant species, they have not been thoroughly investigated in wheat. Here, we identified 34 PIF genes in cultivated hexaploid wheat (Triticum aestivum L). Phylogenetic analysis, exon-intron structures, and protein motif compositions revealed five distinct groups of TaPIFs. Genome-wide collinearity analysis of PIF genes revealed the evolutionary history of PIFs in wheat, Brachypodium distachyon, Oryza sativa, and Arabidopsis thaliana. Cis-regulatory element analysis suggested that TaPIF genes likely participated in plant development and stress responses. Subcellular localization and transcriptional activation assays indicated that most TaPIFs were transcriptionally active and all localized to the nucleus. Gene expression analyses demonstrated that TaPIFs were primarily expressed in the leaves and that they were induced by various biotic and abiotic stresses and phytohormone treatments. This study provides new insights into PIF-mediated stress responses and lays a strong foundation for future investigation of PIF genes in wheat.
**Order of Authors:**	Hua Zhuang
	Zhen Guo
	Jian Wang
	Qing tian Chen, ph.D
**Additional Information:**	
**Question**	**Response**
**Financial Disclosure**<br><br>Enter a financial disclosure statement that describes the sources of funding for the work included in this submission. Review the submission guidelines for detailed requirements. View published research articles from *PLOS ONE* for specific examples.<br><br>This statement is required for submission and **will appear in the published article** if the submission is accepted. Please make sure it is accurate.	This work was supported by the Shaanxi Province "High-level Talents Special Support Plan" Outstanding talents project, Construction of a shared platform for soil quality detection and evaluation in Shaanxi Province (2021PT-053).

Powered by Editorial Manager® and ProduXion Manager® from Aries Systems Corporation

**Unfunded studies**<br>Enter: *The author(s) received no specific funding for this work.*<br><br>**Funded studies**<br>Enter a statement with the following details:<br>• Initials of the authors who received each award<br>• Grant numbers awarded to each author<br>• The full name of each funder<br>• URL of each funder website<br>• Did the sponsors or funders play any role in the study design, data collection and analysis, decision to publish, or preparation of the manuscript?<br>• **NO** - Include this sentence at the end of your statement: *The funders had no role in study design, data collection and analysis, decision to publish, or preparation of the manuscript.*<br>• **YES** - Specify the role(s) played.<br><br>* typeset	
**Competing Interests**<br><br>Use the instructions below to enter a competing interest statement for this submission. On behalf of all authors, disclose any competing interests that could be perceived to bias this work—acknowledging all financial support and any other relevant financial or non-financial competing interests.<br><br>This statement is required for submission and **will appear in the published article** if the submission is accepted. Please make sure it is accurate and that any funding sources listed in your Funding Information later in the submission form are also declared in your Financial Disclosure statement.<br><br>View published research articles from *PLOS ONE* for specific examples.	The authors have declared that no competing interests exist.

**NO authors have competing interests**<br><br>Enter: *The authors have declared that no competing interests exist.*<br><br>**Authors with competing interests**<br><br>Enter competing interest details beginning with this statement:<br><br>*I have read the journal's policy and the authors of this manuscript have the following competing interests: [insert competing interests here]*<br><br>* typeset	
**Ethics Statement**<br><br>Enter an ethics statement for this submission. This statement is required if the study involved:<br><br>• Human participants<br>• Human specimens or tissue<br>• Vertebrate animals or cephalopods<br>• Vertebrate embryos or tissues<br>• Field research<br><br>Write "N/A" if the submission does not require an ethics statement.<br><br>General guidance is provided below. Consult the submission guidelines for detailed instructions. **Make sure that all information entered here is included in the Methods section of the manuscript.**	N/A

**Format for specific study types**<br><br>**Human Subject Research (involving human participants and/or tissue)**<br>• Give the name of the institutional review board or ethics committee that approved the study<br>• Include the approval number and/or a statement indicating approval of this research<br>• Indicate the form of consent obtained (written/oral) or the reason that consent was not obtained (e.g. the data were analyzed anonymously)<br><br>**Animal Research (involving vertebrate animals, embryos or tissues)**<br>• Provide the name of the Institutional Animal Care and Use Committee (IACUC) or other relevant ethics board that reviewed the study protocol, and indicate whether they approved this research or granted a formal waiver of ethical approval<br>• Include an approval number if one was obtained<br>• If the study involved *non-human primates*, add *additional details* about animal welfare and steps taken to ameliorate suffering<br>• If anesthesia, euthanasia, or any kind of animal sacrifice is part of the study, include briefly which substances and/or methods were applied<br><br>**Field Research**<br><br>Include the following details if this study involves the collection of plant, animal, or other materials from a natural setting:<br>• Field permit number<br>• Name of the institution or relevant body that granted permission	
**Data Availability**<br><br>Authors are required to make all data underlying the findings described fully available, without restriction, and from the time of publication. PLOS allows rare exceptions to address legal and ethical concerns. See the PLOS Data Policy and FAQ for detailed information.	Yes - all data are fully available without restriction

Powered by Editorial Manager® and ProduXion Manager® from Aries Systems Corporation

A Data Availability Statement describing where the data can be found is required at submission. Your answers to this question constitute the Data Availability Statement and **will be published in the article**, if accepted.<br><br>**Important:** Stating 'data available on request from the author' is not sufficient. If your data are only available upon request, select 'No' for the first question and explain your exceptional situation in the text box.<br><br>Do the authors confirm that all data underlying the findings described in their manuscript are fully available without restriction?	
**Describe where the data may be found in full sentences. If you are copying our sample text, replace any instances of XXX with the appropriate details.**<br><br>• If the data are **held or will be held in a public repository**, include URLs, accession numbers or DOIs. If this information will only be available after acceptance, indicate this by ticking the box below. For example: *All XXX files are available from the XXX database (accession number(s) XXX, XXX.).*<br>• If the data are all contained **within the manuscript and/or Supporting Information files**, enter the following: *All relevant data are within the manuscript and its Supporting Information files.*<br>• If neither of these applies but you are able to provide **details of access elsewhere**, with or without limitations, please do so. For example:<br><br>*Data cannot be shared publicly because of [XXX]. Data are available from the XXX Institutional Data Access / Ethics Committee (contact via XXX) for researchers who meet the criteria for access to confidential data.*<br><br>*The data underlying the results presented in the study are available from (include the name of the third party*	All relevant data are within the manuscript and its Supporting Information files.

and contact information or URL).<br>• This text is appropriate if the data are owned by a third party and authors do not have permission to share the data.<br><br>* typeset	
Additional data availability information:	Tick here if the URLs/accession numbers/DOIs will be available only after acceptance of the manuscript for publication so that we can ensure their inclusion before publication.

Powered by Editorial Manager® and ProduXion Manager® from Aries Systems Corporation

Manuscript Click here to access/download;Manuscript;Ms-TaPIF-Plos One.doc

Genome-wide identification and comprehensive analysis of the phytochrome-interacting factor (*PIF*) gene family in wheat

Hua Zhuang [1,2], Zhen Guo[1,2], Jian Wang [1,2,3],Tianqing Chen[1,2,3,4,5]*

[1] Shaanxi Provincial Land Engineering Construction Group Co., Ltd, Xi'an, China

[2] Institute of Land Engineering and Technology, Shaanxi Provincial Land Engineering Construction Group Co., Ltd, Xi'an, China

[3] Key Laboratory of Degraded and Unused Land Consolidation Engineering, Ministry of Natural Resources, Xi'an, China.

[4] Shaanxi Engineering Research Center of Land Consolidation, Xi'an, China

[5] Land Engineering Technology Innovation Center, Ministry of Natural Resources, Xi'an, China.

*Corresponding authors: E-mail:369668050@qq.com

Abstract

Phytochrome-interacting factors (PIFs) are essential transcription factors for plant growth, development, and stress responses. Although PIF genes have been well-studied in many plant species, they have not been thoroughly investigated in wheat. Here, we identified 34 PIF genes in cultivated hexaploid wheat (*Triticum aestivum* L). Phylogenetic analysis, exon-intron structures, and protein motif compositions revealed five distinct groups of TaPIFs. Genome-wide collinearity analysis of *PIF* genes revealed the evolutionary history of *PIFs* in wheat, Brachypodium distachyon, Oryza sativa, and Arabidopsis thaliana. Cis-regulatory element analysis suggested that TaPIF genes likely participated in plant development and stress responses. Subcellular localization and transcriptional activation assays indicated that most TaPIFs were transcriptionally active and all localized to the nucleus. Gene expression analyses demonstrated that TaPIFs were primarily expressed in the leaves and that they were induced by various biotic and abiotic stresses and phytohormone treatments. This study provides new insights into PIF-mediated stress responses and lays a strong foundation for future investigation of *PIF* genes in wheat.

Keywords: Wheat; PIFs; phytochrome-interacting factors; expression analysis; abiotic stress

1. Introduction

To allow normal growth and development, plants must sense changes in the wavelength, intensity, direction, and duration of light to which they are exposed [1]. For example, photoperiod is a key influencing factor in the shift from vegetative to reproductive growth. Photoreceptors are critical sensors that enable plants to respond to such light signals. The plant photoreceptors that have been identified to date can be classified as phytochromes (PHYs), cryptochromes (CRYs), UV-B photoreceptors (UVR8s), and phototropins [2-4]. These sensors participate in a range of downstream physiological and biochemical reactions, such as plant growth, stress, and defense responses [2, 5-7]. The photoactivated protein PhyB regulates photomorphogenesis through integration of multiple signaling pathways. It interacts with a set of basic helix-loop-helix (bHLH) transcription factors (TFs) known as PHYTOCHROME INTERACTING FACTOR-LIKE (PIF or PIL) proteins [8, 9]. The first PIF TF to be identified, PIF3 in Arabidopsis thaliana, was discovered in a yeast two-hybrid assay that used the C-terminal region of PhyB as bait [10]. The N-terminal regions of PIF proteins contain a conserved active PhyB binding (APB) motif [11, 12], and the bHLH domain at the C-terminus is responsible for homodimer or heterodimer formation with other bHLHs. The bHLH domain also confers DNA binding activity [13, 14]; previous studies have shown that PIFs directly regulate the expression of downstream genes by binding to G-box (CACGTG) and/or E-box (CANNTG) motifs in the target gene promoters [8].

PIFs have been extensively studied in agronomically important plants, including rice (Oryza sativa) [15], tea [16], and potato [17], each of which have seven known PIFs; maize (Zea mays) [18], tomato [12], and apple [19], which have eight known PIFs each; and Arabidopsis, in which nine PIFs have been identified [8]. PIFs are reportedly crucial regulators of plant growth, development, and stress responses [20-24]. Specifically, PIFs are involved in the regulation of seed germination [25, 26], dark morphogenesis and de-yellowing [27-29], dark-induced senescence [30, 31], flowering time [32, 33], shade avoidance [34], and circadian processes [22]. For example, AtPIF4 and AtPIF5 directly interact with CRYs and bind to target gene promoters in response to blue light exposure [35, 36]. AtPIF4 also

accelerates temperature-mediated entry into the reproductive stage by directly activating the expression of *FLOWERING LOCUS T* (*FT*) [32]. AtPIL5 suppresses seed germination by regulating the expression of genes associated with the gibberellic acid (GA) and abscisic acid (ABA) pathways [8, 37]. AtPIF1 and AtPIF3 negatively regulate the expression of genes related to chlorophyll biosynthesis to influence photomorphogenesis [28, 38]. In rice, PIL15 represses etiolated seedling growth and integrates light and gravitropism signals to regulate tiller angle [39, 40]. PIF4/5 represses *ERF1* expression by directly binding the promoter and negatively regulates plant defense responses to *Botrytis cinerea* through the jasmonic acid (JA) and ethylene (ETH) signaling pathways in *Arabidopsis* [41].

PIF TFs activate or repress the expression of target genes through direct DNA binding and/or interactions with other proteins, often in response to abiotic stressors. As hub proteins that participate in numerous environmental signaling pathways, PIFs can perceive temperature changes, integrate light and temperature signals, and participate in plant temperature signal transduction pathways [42, 43]. For example, AtPIF3 plays a role in hypocotyl growth by regulating freezing tolerance and stomatal opening [44, 45]. Specifically, cold stress facilitates degradation of the F-box proteins AtEBF1 and AtEBF2 to maintain AtPIF3 protein stability. and AtPIF3 also transcriptionally represses the expression of C-repeat binding factors (CBFs) to attenuate plant growth inhibition resulting from low-temperature hypersensitivity [46]. CBFs interact with PIF3, inhibit light-induced degradation of PIF3 and PHYB, and mediate the expression of cold-regulated (COR) and growth-related genes in response to cold stress via the PHYB-PIF signaling pathway [47-49]. AtPIF4 and AtPIF7 are crucial in the early response to thermomorphism and accompany production of growth-promoting hormones [50-53]. PIF1 interacts with ABSCISIC ACID INSENSITIVE 5 (ABI5), a positive regulator of ABA signaling, to influence plant drought tolerance [54, 55]. In maize, PIF3 has roles in water retention and drought resistance; overexpression of ZmPIF1/ZmbHLH165 in rice significantly improves plant water retention [56, 57]. Although PIF functions have been clearly delineated in some plants, systematic identification and functional investigations of PIFs have

primarily been conducted in model plant species, leaving PIFs understudied in agronomically important crops like bread wheat (*Triticum aestivum* L.).

In the present study, a genome-wide analysis of the bread wheat genome was conducted to comprehensively identify *PIF* genes. The identified genes were analyzed to determine their chromosomal locations, gene structures, phylogenetic relationships, stress-inducibility, and the properties of the encoded proteins. To establish the functions of PIFs in wheat, *TaPIF* expression patterns were analyzed in various tissues and under diverse stress conditions using quantitative reverse-transcription PCR (qRT-PCR) and publicly-available transcriptomic data. Finally, transcription activation assays were performed. The results of these experiments provide new insights into the origins, evolutionary history, and functions of *PIFs* in wheat, establishing a solid foundation for future functional characterization of PIFs in this key crop species.

2. Results

2.1. TaPIF identification, classification, and sequence analysis

PIF genes were identified in the wheat genome based on sequence similarity to known AtPIFs. This search yielded a total of 34 TaPIFs (Table 1). Wheat is hexaploid, containing three subgenomes (2n = 6x = 42; AABBDD); we therefore further classified the 34 TaPIFs into 11 homeologous groups. Each gene was named *TaPIF_XA*, *TaPIF_XB*, or *TaPIF_XD*, where X is the homeologous group number and A, B, or D represents the subgenome location. Predicted TaPIF protein length ranged from 157 to 516 amino acids in length (TaPIF4.2-2D and TaPIF7-5D, respectively). The predicted MWs of the proteins ranged from 18.40 kDa to 55.08 kDa (TaPIF4.2-2D and TaPIF7-5D, respectively), and the predicted pI values varied from 5.37 (TaPIF5-5A) to 9.1 (TaPIF4.1-2A, TaPIF4.1-2B, and TaPIF4.1-2D). All of the TaPIFs were predicted to be unstable, with instability indexes > 53.56; had negative GRAVY scores, indicating that they were hydrophilic; and were predicted to be localized to the nucleus.

Table 1. Characterization of PIFs in wheat.

2.2. Phylogenetic and syntenic analyses

To analyze the relationships between PIF family members, a phylogenetic tree was constructed with the 34 TaPIFs and 52 PIFs from *Arabidopsis*, *Z. mays*, *O. sativa*, and *B. distachyon* (Figure 1; Table S1). The PIF proteins from these five species were clustered into five groups (I, II, III, IV, and V). TaPIFs were distributed among all five groups, whereas neither OsPIFs nor ZmPIFs were present in group V. TaPIF-6A/B/D and TaPIF-9A/B/D clustered with BdPIF6 and BdPIF9, respectively, in group V; TaPIF-7A/B/D and TaPIF-8A/B/D clustered with ZmPIF7 and ZmPIF8, respectively, in group II; and TaPIF-10A/B/D and TaPIF-11A/B/D clustered with BdPIF10 and BdPIF11, respectively, in group III (Fig 1A).

Fig 1. Phylogenetic analysis of PIF families in *Arabidopsis thaliana*, rice, maize, *Brachypodium distachyon*, and wheat.

Similarities in gene or protein sequences can be used to annotate a newly-sequenced genome based on the annotations of homologs in a well-established, previously annotated genome. In wheat, all of the *TaPIF* genes could be clustered into 11 homoeologous groups. For example, *TaPIF-1A*, *TaPIF-1B*, and *TaPIF-1D* all belonged to the *TaPIF-1* homoeologous group. Most duplication events in the wheat genome have been between copies of a single chromosome in different subgenomes, but there have also been inter-chromosomal gene replication events, such as those between chromosome 2 and chromosome 5 (Figure 2A). This phenomenon was here illustrated by a collinear relationship between *TaPIF3* on chromosomes 2A and 2D and *TaPIF8* on chromosomes 5A and 5B. The event from which these duplicates were derived resulted in at least 37 pairs of collinear relationships in the wheat genome; this included 34 collinear pairs between a single chromosome in different subgenomes and three collinear pairs between chromosome 2 and chromosome 5 (Fig 2A; Table S2).

Fig 2. *PIF* synteny analysis.

To further understand the evolutionary relationships between members of the TaPIF family,

collinearity analysis was conducted with *O. sativa*, *Arabidopsis*, and *B. distachyon*. Wheat showed seven, 25, and 37 homologous *PIF* gene pairs, respectively, with these species (Fig 2B; Table S2). Notably, chromosomes 5A/B/D showed the highest homology with other species. There were several collinear blocks between wheat and the dicot species *Arabidopsis* (namely *AtPIL1* and *TaPIF1-1A/D*; *AtLRL2* and *TaPIF9-6B/D*; and *AtLRL3* and *TaPIF6-5/B/D).* However, Ka/Ks values could not be calculated for these gene pairs due to the high level of sequence divergence (Table S2). In rice and *B. distachyon*, PIF genes had been subject to strong purifying selection, and we found the largest number of gene duplication events between *B. distachyon* and wheat. Specifically, *TaPIF-2A/D* was collinear with *OsPIL16*; *TaPIF5-5A/D* and *TaPIF7-5A/B/D* were collinear with *OsPIL11*; and *TaPIF8-5A/B* was collinear with *OsPIL14*. There were as many as 37 homeologous gene pairs between *B. distachyon* and wheat, for example *TaPIF6-5A/B/D* with *BdPIF6/12* and *TaPIF7-5A/B/D* with *BdPIF7/8*.

2.3. *TaPIF* gene structure and conserved protein motifs

To investigate structural diversity among *TaPIF* genes, a second phylogenetic tree was constructed using only the 34 full-length TaPIF sequences (Figure 1a). Diversity in exon-intron structure often plays a pivotal role in the evolution of gene families, and orthologous genes tend to have highly conserved gene structures; these data can offer additional evidence to reveal or support phylogenetic groupings and evolutionary relationships [73, 74]. We therefore mapped exon-intron organization in the coding regions of each *TaPIF* gene. This revealed that each *TaPIF* contained between from four to seven exons. Specifically, *TaPIF1*, *TaPIF5*, and *TaPIF7* had four exons; *TaPIF2* and *TaPIF4* had five exons; *TaPIF9* and *TaPIF11* had six exons; and the remaining *TaPIFs* had seven exons (Fig 3).

Fig 3. Phylogenetic relationships, conserved motifs, and gene structures of TaPIFs.

To further characterize structural diversity and determine TaPIF functions, the MEME suite was used to identify 10 motifs among the TaPIFs (Fig 3, Table S3, and Fig S1). The most closely-related family

members (as determined from the phylogenetic analysis) showed similar motif arrangements and positions, indicating that PIFs within each group may have performed similar biological functions. All of the TaPIFs contained the same arrangement of motifs 1, 2, and 5. Conservation of some motifs between groups suggested that these proteins may all have similar functions, although the presence of group-specific domains and motifs would allow for functional specialization.

2.4. *Cis*-acting element analysis

Gene transcription is regulated by *cis*-acting elements that function as TF binding sites [75]. Analysis of upstream regulatory sequences can therefore offer clues regarding the regulatory patterns and potential functions of target genes. Here, we conducted *cis*-element analysis in the 2-kb region upstream of the translation start site in each TaPIF. This revealed the presence of cis-elements involved in growth, development, stress responses, and hormone responses (Fig 4; Table S4). The *TaPIF* promoters contained many hormone-related motifs, including auxin response elements (TGA-elements and AuxRE-cores), GA response elements (P-boxes, GARE-motifs, and TATC-boxes), ABA response elements (ABREs), SA response elements (TCA-elements), and methyl jasmonate (MeJA) response elements (TGACG-motifs and CGTCA-motifs). ABA- and MeJA-response elements were the most abundant hormone-related cis-elements; these were present in the promoters of every *TaPIF*. In addition, the *TaPIF11-7B* promoter contained at least one *cis*-element responsive to each of the hormone classes described above, indicating that this gene may play a crucial role in the hormonal response regulatory network.

Fig 4. Analysis of *TaPIF* promoter *cis*-elements.

A large number of stress-related cis-elements were also found in TaPIF promoters. As expected, light-response elements (G-boxes, TCT-motifs, I-boxes, Sp1s, and MREs) were found in the promoters of all *TaPIF* genes and were the most abundant class of cis-elements. The promoters of

21 *TaPIFs* contained drought-inducible MYB binding sites (MBSs) or dehydration-responsive elements (DREs), suggesting that these genes may be involved in wheat drought responses. Low-temperature responsive (LTR) elements, another major type of stress-related motif, were found in the promoters of 12 *TaPIF* genes. Six *TaPIFs* contained the wound-responsive WUN-motif, and seven *TaPIFs* contained TC-rich repeats, which are involved in defense and stress responses. Notably, some of the identified cis-elements were involved in organ- or tissue-specific processes, such as endosperm expression (GCN4 motif), meristem expression (CAT-box), root-specific expression (motif I), seed-specific regulation (RY-element), cell cycle regulation (MSA-like), and zein metabolism regulation (O2-site) (Fig 4; Table S4). Interestingly, the *TaPIF3-2D* and *TaPIF9-6B* promoter regions contained endosperm expression and zein metabolism regulation elements, suggesting that these genes may play key roles in wheat seed development. These results demonstrated that *TaPIF* genes may be involved in plant development and in hormone and stress responses.

2.5. Tissue-specific *TaPIF* expression

RNA-seq is an effective method for investigating transcriptional patterns of specific genes in a high-throughput manner [76]. To investigate the potential biological functions of TaPIFs, RNA-seq data were analyzed to identify TaPIF expression patterns during wheat growth and development. The analyzed tissues were the roots, stems, leaves, flag leaves, spikes, and grains. Systematic clustering analysis was performed for all 34 *TaPIFs* based on their spatiotemporal expression patterns (i.e., expression in various tissues and at different developmental timepoints) (Fig 5; Table S5). In general, *TaPIFs* were expressed in many tissue types across multiple developmental stages. TaPIFs were expressed at particularly high levels in the stems, flag leaves, and young spikes. Specifically, *TaPIF3*, *TaPIF5*, *TaPIF7*, and *TaPIF8* were highly expressed in the leaves, flag leaves, and young spikes; TaPIF4 was highly expressed in the grains; *TaPIF6* was most highly expressed during vegetative

growth, especially in the roots; and *TaPIF10* was expressed at the highest levels in the stems and at the early grain stage. *TaPIF11* was expressed at relatively low levels throughout all developmental stages. These expression patterns indicated that *TaPIFs* likely have strongly influential functions in wheat development.

Fig 5. Tissue-specific *TaPIF* expression patterns

2.6. *TaPIF* responses to biotic stress

To understand the roles of PIFs under biotic stress conditions, we analyzed TaPIF expression patterns in wheat infected with stripe rust or powdery mildew (Fig 6, Table S5). *TaPIF1-1A/B/D*, *TaPIF7-5A/B/D*, and *TaPIF8-5A/D* were significantly up-regulated at 24 h of treatment with stripe rust; in contrast, TaPIF5-5A/B/D, TaPIF9-6B, and *TaPIF10-6A* were strongly down-regulated at 24 h after stripe rust infection. There were no significant changes in expression levels of *TaPIF4A/B/D*, *TaPIF6A/D*, *TaPIF9-6A/D*, *TaPIF10-6B/D*, or *TaPIF11A/B/D* between 0 and 72 h. *TaPIF3-2A/B/D*, *TaPIF5-5A/B/D*, *TaPIF7-5A/B/D* were up-regulated in response to powdery mildew treatment, whereas *TaPIF2-1A/B/D* were down-regulated. There were no significant differences in expression levels of *TaPIF1-1A/D*, *TaPIF4s*, *TaPIF6-5A/B/D*, *TaPIF9-6A/B/D*, or *TaPIF10-6A/B/D* (Fig 6, Table S5). These results indicated both similarities in stress responses among some TaPIFs and diversity in expression profiles between other family members.

Fig 6. *TaPIF* expression in response to biotic stress.

2.7. *TaPIF* responses to abiotic stress and hormone treatment

To explore potential functions of PIFs in abiotic stress responses, we analyzed *TaPIF* expression patterns in wheat treated with drought, heat, cold, and salt stress treatments (Fig 7A and B, Table S5). Under drought conditions, *TaPIF1-1A/B/D*, *TaPIF6-5D*, *TaPIF9-6D*, and *TaPIF10-6A/B/D*

were up-regulated compared with control plants; there were no significant changes in *TaPIF4* expression levels, and the remainder of *TaPIF* genes were down-regulated (Fig 7A, Table S5). Under heat stress, *TaPIF6-5A/D* and *TaPIF8-5A/D* were up-regulated, whereas all other TaPIFs were down-regulated (Fig 7A, Table S5). In response to salt stress, *TaPIF3-2A/D*, *TaPIF6-5A/B/D*, and *TaPIF8-5A* were up-regulated, and *TaPIF1-1D*, *TaPIF5-5B/D*, *TaPIF7-5A/B/D*, *TaPIF9-6A/B/D*, and *TaPIF10-6A/B/D* were down-regulated. *TaPIF1-1A/B/D*, *TaPIF2-1A/B/D*, *TaPIF9-6B/D*, and *TaPIF10-6A/B/D* were up-regulated under cold conditions, whereas *TaPIF3-2A/B/D*, *TaPIF5-5A/B/D*, *TaPIF6-5A/B/D*, *TaPIF7-5A/B/D*, and *TaPIF8-5D* were down-regulated (Fig 7B, Table S5). These results again showed both commonalities and diversity in *PIF* responses to stressors.

Fig 7. Gene expression patterns of *TaPIF*s in response to abiotic stress or phytohormone treatment.

Plant hormones are involved in all aspects of plant development. We therefore explored PIF expression patterns in response to treatment with the phytohormones ABA, 6-BA, SA, GA, and JA (Fig 7C, Table S5). All *TaPIFs* were down-regulated in response to SA treatment. *TaPIF2-1A/B/D* and *TaPIF4.1-2A/B* were down-regulated under both ABA and 6-BA treatments; *TaPIF1-1A/B/D*, *TaPIF3-2A/B/D*, *TaPIF6-5A/B/D*, *TaPIF8-5A/B/D*, and *TaPIF10-6A/B/D* were down-regulated in response to 6-BA only; and *TaPIF3-2A/B/D*, *TaPIF5-5A/B/D*, *TaPIF7-5A/B/D*, and *TaPIF11-7A* were up-regulated in response to ABA exposure (Fig 7C, Table S5). Overall, *PIFs* had divergent responses to ABA, 6-BA, SA, GA, and JA, suggesting that the *TaPIFs* differed in hormonal pathway membership.

2.8. *TaPIF* responses to PEG and ABA treatment

To investigate whether *TaPIF* genes were drought-responsive, we used qRT-PCR to examine *TaPIF*

expression in the leaves of plants treated with PEG-induced osmotic stress. Because ABA is a signal transducer in the drought stress response pathway, we also analyzed the effects of ABA treatment on *TaPIF* expression. Allele pairs from the A-, B-, and D-subgenomes were grouped and tested together due to the high sequence similarity in the corresponding transcripts. The analyzed genes exhibited highly divergent responses to the drought stress treatments (Fig 8). *TaPIF1* and *TaPIF2* were up-regulated in response to PEG treatment, especially at 12 h; this indicated the high responsiveness of these two genes to osmotic stress. *TaPIF3*, TaPIF7, and *TaPIF9* showed highly similar expression profiles in response to osmotic stress. *TaPIF4*, *TaPIF8*, and *TaPIF11* were down-regulated to similar degrees. *TaPIF6* and *TaPIF10* were slightly up-regulated at the first few timepoints after the onset of osmotic stress, suggesting that they may have roles in the early osmotic stress response. At 1 h after ABA treatment, *TaPIF1*, *TaPIF2*, *TaPIF4*, *TaPIF8*, *TaPIF9*, and *TaPIF10* were significantly up-regulated, and *TaPIF3* and *TaPIF7* were up-regulated after 6 h. These expression patterns indicate that *TaPIFs* may play an important role in osmotic stress by ABA dependent pathway.

Fig 8. Expression profiles of *TaPIFs* treated with polyethylene glycol (PEG) or abscisic acid (ABA).

2.9. TaPIF transactivation activity and subcellular localization

We next sought to isolate and functionally characterize several *TaPIFs*. Transactivation assays were performed in yeast with three TaPIFs, .Each gene was cloned from the wheat cultivar ‘Chinese Spring’, inserted into the vector pGBKT7, and transformed into yeast. TaPIF2 and TaPIF5 promoted increased yeast growth on SD/-Trp/-His/-Ade medium compared to yeast transformed with the empty vector negative control, while TaPIF10 were not grow well on SD/-Trp/-His/-Ade medium same as

empty vector control (Fig 9A). The α-galactosidase activity further supported these results. These results revealed that TaPIF2 and TaPIF5, instead of TaPIF10 proteins had transactivation activity.

Fig 9. Subcellular localization and transactivation activity of TaPIFs in wheat.

Sequence analysis had predicted that each of the TaPIFs would be localized to the nucleus (Table 1). To validate these subcellular localization predictions, we developed transient expression vectors to express plasmids encoding TaPIF-GFP fusion proteins (TaPIF2-GFP, TaPIF5-GFP, and TaPIF10-GFP) in wheat protoplasts. As expected, visualization of transformed cells showed that all three TaPIF-GFP fusion proteins were localized to the nucleus (Fig 9B).

3. Discussion

3.1. Identification and evolutionary analysis of the PIF family

PIF proteins comprise a subfamily of the bHLH TF superfamily [37]. Previous functional characterizations have shown that these proteins are involved in plant growth, development, and stress responses [77]. In the present study, we identified 34 *PIF* genes in wheat (Table 1), which is more PIF genes than in the Arabidopsis, rice, maize, or B. distachyon genomes (Table S1). Because heterohexaploid wheat has three subgenomes, the 34 *TaPIFs* formed 11 homeologous groups. Polyploidization events (such as those leading to heterohexaploid wheat), along with whole-genome duplication (WGD) and amplification events, play major roles in shaping genome evolution in many plant species, including upland cotton (*Gossypium hirsutum*) and Brassica napus [78]. Indeed, gene family expansion is primarily driven by gene duplication events, including WGD, segmental duplication, and tandem duplication events [79]. Here, collinearity analysis of wheat, *O. sativa*, *Arabidopsis*, and *B. distachyon* revealed 37, seven, 27, and 37 duplicate PIF gene pairs, respectively (Fig 2; Table S2). Although there were several collinear genes between wheat and the dicot species *A. thaliana* (namely *AtPIL1* and *TaPIF1-1A/D*; *AtLRL2* and *TaPIF9-6B/D*; and *AtLRL3* and *TaPIF6-5/B/D*), the associated Ka/Ks values could not be calculated due to high level of sequence divergence (Table S2). In the monocot species rice and B. distachyon, *PIF* genes showed evidence of

purifying selection. Additionally, there had been more gene duplication events in B. distachyon and wheat compared to rice. These results demonstrated that WGD/segmental duplication was the predominant factor causing expansion of the PIF gene family in wheat and other species.

Phylogenetic analysis revealed that PIFs present in the five analyzed plant species could be classified into five subgroups (Fig 1A). Most PIF proteins in monocots (wheat, rice, maize, and *B. distachyon*) were distributed among groups II and III, whereas most of the PIFs in the dicot *Arabidopsis* were distributed in groups IV and V (Fig 1B). These results demonstrated sequence diversity between PIFs in monocot compared to dicot species (Fig 1). Analysis of gene structure and protein motifs showed that the TaPIFs within each subclade shared similar protein lengths, gene structures, and motif compositions; this implied relatively high evolutionary conservation among TaPIFs (Fig 3).

3.2. *TaPIF* expression was tissue-specific

In several crop plants, *PIF* expression has previously been shown to be largely tissue-specific and to strongly influence key developmental characteristics. For example, in rice, the PIF gene *OsPIL13* is highly expressed in the node portions of the stem, and overexpression of the gene promotes internode elongation and reduces plant height [80]. In maize, *ZmPIF1* and *ZmPIF3* are highly expressed in the pistils and leaves, and these genes enhance grain yield by increasing tiller and panicle number [57, 81]. In tomato (Solanum lycopersicum), *SlPIF4* is highly expressed in the leaves and fruits, but is significantly down-regulated after ripening [82]. We here found that the majority of *PIFs* were most highly expressed in the stems and leaves (particularly in the flag leaves), and were expressed at lower levels in the grain (Fig 5). However, some genes did not follow this pattern; TaPIF4s were specifically expressed in the grain, and *TaPIF6-5A*/B were expressed at significantly higher levels in the root (Figure 6). Spatiotemporal differences in expression of these genes may be related to wheat growth and developmental processes.

3.3. TaPIFs played important roles in hormone signaling pathways

In *Arabidopsis*, PIFs are known to be involved in multiple hormone signaling pathways [83]. AtPIF1 controls seed germination by regulating the ABA and GA signaling pathways [55, 84]. AtPIF4 and AtPIF5 can integrate light signals and the auxin signaling pathway to regulate plant growth via circadian rhythm [85]. Similarly, we here found that most wheat *PIF* genes contained at least one hormone-responsive cis-element in the promoter region (Fig 4, Table S4). *TaPIF2-1A/B/D* and *TaPIF4.1-2A/B* were down-regulated in response to treatment with either ABA or 6-BA; *TaPIF1-1A/B/D*, *TaPIF3-2A/B/D*, *TaPIF6-5A/B/D*, *TaPIF8-5A/B/D*, and *TaPIF10-6A/B/D* were down-regulated by 6-BA treatment; and *TaPIF3-2A/B/D*, *TaPIF5-5A/B/D*, *TaPIF7-5A/B/D*, and *TaPIF11-7A* were up-regulated by ABA (Fig 7C, Table S5). Notably, some of the homologous *PIF* gene pairs showed differing expression patterns in response to ABA, SA, JA, or GA treatment. For example, *TaPIF3-2A* and *TaPIF8-5D* were down-regulated in response to JA, whereas *TaPIF3-2B* and *TaPIF8-5B* were up-regulated. In response to GA treatment, *TaPIF11-7A/D* and *TaPIF11-7B* had opposite expression patterns (Fig 7C; Table S5); the same was true of *TaPIF7-5A/D* and *TaPIF7-5B* in plants treated with ABA. These results suggested that *PIFs* may have been involved in the regulatory network that governs responses to numerous hormones in wheat, thus affecting growth and defense responses.

3.4. *TaPIFs* responded to multiple abiotic stresses

Cis-acting elements in the promoter region play important roles in regulating gene expression. When plants suffer from various abiotic environmental stresses, such as cold, drought, or salinity, signaling cascades activate TFs. These TFs then regulate the expression of downstream target genes through binding to specific *cis*-elements [86-90]. We found *cis*-elements related to three main categories in the promoter regions of *TaPIF* genes: development and metabolism, hormone responses, and stress responses (Figure 4). These results suggested critical roles of *TaPIF* genes in plant development and stress responses. Furthermore, gene expression analysis revealed diversified expression patterns of TaPIFs, with specific genes showing unique spatiotemporal expression profiles (Figure 5, Figure 6,

and Figure 7). The varied expression patterns indicated functional diversification of *TaPIF* roles in wheat growth and development.

Notably, *TaPIF2*, *TaPIF3*, *TaPIF5*, *TaPIF7*, and *TaPIF8* were shown to be significantly differentially expressed in response to powdery mildew and stripe rust infection. *TaPIF5-5A/B/D* was up-regulated after stripe rust infection and down-regulated in response to powdery mildew, and similar expression patterns occurred for *TaPIF7* and TaPIF8. A key component of pathogen defense is phytohormone crosstalk, in which SA and JA have major roles [91-93]. Interestingly, *TaPIF2, TaPIF3, TaPIF5, TaPIF7*, and *TaPIF8* showed distinct responses to SA and JA (Figure 6), consistent with the expression profiles of known pathogen-responsive genes. *TaPIF6-5D* was up-regulated under drought, salt, and heat stress conditions, suggesting that it may have a critical function in wheat during responses to multiple abiotic stresses. *TaPIF1-1A/B/D* expression was induced by both cold and drought stresses, and TaPIF8-5A responded to both salt and cold stress. Furthermore, all 34 *TaPIFs* were differentially expressed (to varying degrees) in the leaves in response to PEG-induced simulated drought or ABA treatment (Figure 8). The functionalities suggested by the expression profiles were similar to the known functions of PIFs in other crop species, such as rice and maize [32, 56, 94]. Overall, these results serve as a key reference point for future validation of TaPIF functions.

3.5. TaPIFs functioned in the nucleus

In vitro transcriptional activity assays with three *TaPIFs*, provided empirical evidence that at least some TaPIFs serve as transcriptional activators (Fig 9A). Furthermore, subcellular localization assays confirmed that the three TaPIFs were localized to the nucleus as predicted (Fig 9B). In *Arabidopsis* [95-97], rice [80, 98, 99], maize [56, 57], and other plants [100-103], PIFs function as TFs that transcriptionally regulate downstream target genes during normal growth, development,

and stress responses. They perform this function either alone or through interactions with other proteins. For example, the phyB-DET1-HFR1-PIF1 signaling pathway regulates seed germination in response to light in *Arabidopsis* [95]; PIF4/TCP4–KRP1 restricts cell division and leaf size in response to high temperature [96]; PIF4 transcriptionally represses *SPCH* under high-temperature conditions, resulting in stomatal development [97]; and the FLS2–RBOHD–PIF4 module regulates responses to drought and salt stress in Arabidopsis [104]. In rice, OsPIL16 positively regulates *OsDREB1* expression to enhance cold tolerance [105], and OsPIL13 and OsPIF14 induce growth by regulating cell wall organization and cell elongation genes in response to drought and salt stress [80, 99, 106]. Together, these results indicate that PIFs act as key integrators in biotic and abiotic stress signaling pathways that regulate plant growth.

4. Materials and Methods

4.1. Plant materials and abiotic stress treatments

T. aestivum cv. 'Chinese Spring' was used in all experiments in this study. Seeds were surface-sterilized with 0.1% sodium hypochlorite, then washed with deionized water. After sterilization, seeds were placed on wet filter paper and incubated at 24°C for 4 d to germinate. Germinated seeds were moved to a light incubator and grown at 15°C under a 16/8 h light/dark cycle in Hoagland's nutrient solution as previously described [58].

Simulated drought treatments were conducted in seedlings at the three-leaf stage by immersing the roots in Hoagland's nutrient solution containing 20% polyethylene glycol (PEG)-6000. ABA treatments were carried out the same way, but with 100 μM ABA instead of PEG. For both treatments, leaf samples were collected at 0, 1, 3, 6, 12, 24, and 48 h and washed. Collected samples were snap-frozen in liquid nitrogen, then stored at -80°C prior to RNA extraction.

4.2. Genome-wide *PIF* identification in wheat and gene annotation

PIF genes were identified in the wheat genome using the IWGSC RefSeq v1.1 reference genome assembly (https://wheat-urgi.versailles.inra.fr/). The sequences of all 18 known Arabidopsis PIFs were downloaded from The Arabidopsis Information Resource (TAIR) (http://www.arabidopsis.org/) and used as queries in BLASTP searches of the wheat genome. Redundant sequences were removed from the results, then the presence of the conserved HLH domain (IPR011598) was verified in the remaining sequences using SMART (http://smart.embl.de/). The biochemical properties of the resulting TaPIF proteins were then examined. These properties comprised the grand average of hydropathicity (GRAVY), molecular weight (MW), theoretical isoelectric point (pI), and instability index, which were predicted with ExPasy (http://web.expasy.org) [59]. The subcellular localization of each protein was predicted using Cell-PLoc 2.0 (http://www.csbio.sjtu.edu.cn/bioinf/plant-multi/) [60].

4.3. TaPIF phylogenetic and synteny analyses

The sequences of known PIF proteins in Arabidopsis, maize, rice, and Brachypodium distachyon were obtained from JGI (https://phytozome.jgi.doe.gov/pz/portal.html). These proteins combined with the TaPIFs totaled 86 sequences. ClustalW [61] was used with default settings to align the full-length amino acid sequences. A phylogenetic tree was constructed from the aligned sequences using the Neighbor-Joining (NJ) method with 1000 bootstrap replicates in MEGA7 [62]. The resulting phylogenetic tree was visualized with iTOL (https://itol.embl.de/personal_page.cgi). Among the TaPIFs, duplicate gene pairs were identified using the TBtools program MCScanx with an E-value threshold of $< 1 \times 10^{-10}$. Genes were classified as tandem duplicates when adjacent homologous TaPIF genes were present on a single chromosome with no more than one gene separating them. Genes were considered to have been derived from segmental duplication events where there was evidence of polyploidy or chromosome rearrangement. Collinear relationships between *TaPIFs* and *PIF* genes in *Arabidopsis* and *B. distachyon* were also analyzed with MCScanx using the default parameters. TaPIF chromosomal locations, duplications, and collinearity were visualized in Circos [63] and TBtools [64].

4.4. Exon-intron structure and conserved motif analyses

The exon-intron structures of *TaPIFs* were determined based on the reference genome annotations. TBtools [64] was used to visualize the gene structures. The MEME suite (v5.3.3) was used to identify conserved protein motifs using the following parameters: number of unique motifs, 10; motif length, 6~50 amino acids; number of non-overlapping occurrences of each motif in a single sequence, any.

4.5. *Cis*-acting element analysis

To determine potential functions of the *TaPIF* genes, we performed cis-regulatory element analysis of the promoter regions. For each gene, the 2-kb region upstream of the start codon was analyzed with the PlantCARE database (http://bioinformatics.psb.ugent.be/webtools/plantcare/html/) [65]. A heatmap constructed with TBtools [64] was used to visualize the distribution of key types of cis-acting elements among the *TaPIF* genes.

4.6. RNA-sequencing (RNA-seq) data analyse

Spatiotemporal TaPIF expression profiles were analyzed using publicly-available expression data downloaded from WheatOmics 1.0 (http://202.194.139.32/) and WheatExp (https://wheat.pw.usda.gov/WheatExp/) [66-67]. *TaPIF* responses to biotic stress conditions were analyzed using RNA-seq datasets for wheat infected with stripe rust (*Puccinia striiformis f. sp. Tritici*) or powdery mildew (*Blumeria graminis f. sp. Tritici*) (http://202.194.139.32/expression/index.html) [68]. TaPIF responses to abiotic stress conditions and hormone treatments were analyzed using RNA-seq datasets for wheat treated with drought, heat, cold, salt, 6-benzylaminopurine (6-BA), ABA, GA, JA, and salicylic acid (SA) (http://202.194.139.32/expression/index.html) [67, 69, 70]. For all RNA-seq datasets, expression levels were calculated in fragments per kilobase of transcript per million mapped reads (FPKM) and visualized with heatmaps generated in TBtools.

4.7. RNA extraction and qRT-PCR

Expression levels of *TaPIF* genes were analyzed in response to simulated drought stress and ABA treatment as described above (section 4.1). The RNAprep Pure Plant Kit (TIANGEN BIOTICH (BEIJING) CO., LTD, DP432) was used to isolate total RNA from leaf tissue following the manufacturer's instructions. cDNA was synthesized from the resulting RNA with the HiScript III 1st Strand cDNA Synthesis Kit (+gDNA wiper) (Vazyme Biotechnology Co., Ltd, R312). Each qRT-PCR reaction system was 20 μL in total, comprising 10 μL of 2×ChamQ SYBR Color qPCR Master Mix (Vazyme Biotechnology Co., Ltd, Q411), 2 μL of template cDNA, 0.4 μL each forward and reverse primers (10 μM), and 7.2 μL of ddH2O. PCR was performed in a CFX 96 Real-Time PCR Detection System (Bio-Rad, USA) with the following program: 30s at 95°C (pre-denaturation); 40 cycles of 10 s at 95 °C and 30 s at 60 °C. Mean expression values were calculated from three biological replicates. TaPIF expression levels were normalized to the internal control gene TaActin1 with the $2^{-\Delta\Delta Ct}$ method [71]. All qRT-PCR primers are shown in Table S6.

4.8. Subcellular localization analysis

TaPIF–green fluorescent protein (GFP) fusion proteins were generated to determine TaPIF subcellular localization. The coding regions of *TaPIF2*, *TaPIF5*, and *TaPIF10* were amplified, then each was separately fused to the region encoding the N-terminal end of GFP in the CaMV35S-GFP-NOS vector. Leaf tissue was collected from 2-week-old wheat seedlings and protoplasts were isolated as previously described [72]. The resulting protoplasts were transformed with a TaPIF-GFP plasmid or the empty vector using a PEG-mediated transfection protocol [72]. Transfected protoplasts were incubated in W5 solution (comprising 2 mM MES, 154 mM NaCl, 125 mM CaCl2, and 5 mM KCl at pH 5.7) in the dark at 23 °C for 18 h. Fluorescent signals were visualized with an FV3000 laser-scanning confocal microscope (OLYMPUS, Japan).

4.9. Subcellular localization analysis

The full coding sequences of *TaPIF2*, *TaPIF5*, and *TaPIF10* were amplified via PCR and each was inserted into the pGBKT7 vector. Chemically competent AH109 yeast cells (Shanghai Weidi

Biotechnology Co., Ltd, YC1010) were transformed with the TaPIF construct or empty vector, plated on SD/-Trp media, and incubated for 3 d at 30°C. Transformants were streaked onto several types of dropout media: SD/-Trp, SD/-Trp/-His/-Ade, and SD/-Trp/-His/-Ade/x-α-gal. The transactivation activity of each protein was evaluated based on yeast cell growth

5. Conclusions

Throughout the world, wheat yield is severely threatened by extreme environmental conditions. Drought and high temperatures in particular are major stressors that limit grain production. It is critical to understand the functions and interactions of genes involved in stress responses to enable identification of genetic solutions for increased wheat yield. In the present study, we identified 34 *PIF* genes in wheat and analyzed their phylogeny, gene structure, protein motif composition, collinear relationships, and expression patterns. The results of this comprehensive *TaPIF* characterization indicated that PIFs are pivotal players not only in wheat development but also in stress responses. Future studies should continue to explore PIF functions and identify candidates for cloning and genetic manipulation to aid plants in coping with ever-increasing environmental challenges.

Author contributions

Tianqing Chen and Hua Zhuang conceived and designed the study. Zhen Guo and Jian Wang performed the experiments. Hua Zhuang and Zhen Guo analyzed the data, wrote the manuscript, and revised the manuscript. All authors have read and approved the final version of the manuscript.

Conflicts of interest

The authors declare no conflict of interest.

Acknowledgments

This work was supported by the Shaanxi Province "High-level Talents Special Support Plan" Outstanding talents project, Construction of a shared platform for soil quality detection and evaluation in Shaanxi Province (2021PT-053).

Reference

1. Hoang Q. Han Y, Kim J. Plant Phytochromes and their Phosphorylation. International journal of molecular sciences 2019; 20: 3450.
2. Cashmore A, Jarillo J, Wu Y, Liu, D. Cryptochromes: blue light receptors for plants and animals. Science (New York, N.Y.) 1999; 284:760-764.
3. Quail P. Phytochrome photosensory signalling networks. Nature reviews. 2002; 3: 85-93.
4. Li F, Mathews S. Evolutionary aspects of plant photoreceptors. Journal of plant research. 2016; 129, 115-122.
5. Ballaré C. Light regulation of plant defense. Annual review of plant biology. 2014, 65:335-363.
6. Gururani M, Ganesan M, Song P. Photo-biotechnology as a tool to improve agronomic traits in crops. Biotechnology advances. 2015; 33: 53-63.
7. Chen M, Chory J. Phytochrome signaling mechanisms and the control of plant development. Trends in cell biology. 2011; 21:664-671.
8. Leivar P, Quail P. H. PIFs: pivotal components in a cellular signaling hub. Trends in Plant Science. 2011; 16: 19-28.
9. Heim M, Jakoby M, Werber M, Martin C, Weisshaar B, Bailey P. The basic helix-loop-helix transcription factor family in plants: a genome-wide study of protein structure and functional diversity. Molecular biology and evolution. 2003; 20:735-747.
10. Ni M, Tepperman J, Quail P. PIF3, a phytochrome-interacting factor necessary for normal photoinduced signal transduction, is a novel basic helix-loop-helix protein. Cell. 1998; 95:657-667.
11. Khanna R, Huq E, Kikis E, Al-Sady B, Lanzatella C, Quail P. A novel molecular recognition motif necessary for targeting photoactivated phytochrome signaling to specific basic helix-loop-helix transcription factors. The Plant cell. 2004; 16:3033-3044.
12. Lee N, Choi G. Phytochrome-interacting factor from *Arabidopsis* to liverwort. Current opinion in plant biology. 2017; 35:54-60.
13. Bu Q, Castillon A, Chen F, Zhu L, Huq E. Dimerization and blue light regulation of PIF1 interacting bHLH proteins in Arabidopsis. Plant molecular biology. 2011; 77: 501-511.
14. Hornitschek P, Lorrain S, Zoete V, Michielin O, Fankhauser C. Inhibition of the shade avoidance response by formation of non-DNA binding bHLH heterodimers. The EMBO journal. 2009; 28:3893-3902.
15. Nakamura Y, Kato T, Yamashino T, Murakami M, Mizuno T. Characterization of a set of phytochrome-interacting factor-like bHLH proteins in Oryza sativa. Bioscience, biotechnology, and biochemistry. 2007; 71: 1183-1191.
16. Zhang X, Xiong L, Luo Y, Wen B, Wang K, Liu Z, et al. Identification, molecular characteristic, and expression analysis of PIFs related to chlorophyll metabolism in tea plant (*Camellia sinensis*). International journal of molecular sciences. 2021; 22:10949.
17. Jian H.J, Shang L.N, Jin Z.H, Ding Y, Yan L.I, Wang J.C, et al. Genome-wide identification and

characterization of *PIF* genes and their response to high temperature stress in potato. Acta Agronomica Sinica. 2021;48:86-98.

18. Kumar I, Swaminathan K, Hudson K, Hudson M. Evolutionary divergence of phytochrome protein function in Zea mays PIF3 signaling. Journal of experimental botany. 2016; 67: 4231-4240.
19. Zheng P, Wang X, Yang Y, You C, Zhang Z, Hao Y. Identification of Phytochrome-interacting factor family members and functional analysis of of MdPIF4 in *Malus domestica*. International journal of molecular sciences. 2020; 21:7350.
20. Kudo M, Kidokoro S, Yoshida T, Mizoi J, Todaka D, Fernie A, et al. Double overexpression of DREB and PIF transcription factors improves drought stress tolerance and cell elongation in transgenic plants. Plant biotechnology journal. 2017;15, 458-471.
21. Peng M, Li Z, Zhou N, Ma M, Jiang Y, Dong A, et al. Linking PHYTOCHROME-INTERACTING FACTOR to histone modification in plant shade avoidance. Plant physiology. 2018; 176:1341-1351.
22. Leivar P, Monte E. PIFs: systems integrators in plant development. The Plant cell. 2014; 26: 56-78.
23. Dong J, Tang D, Gao Z, Yu R, Li K, He H, et al. *Arabidopsis* DE-ETIOLATED1 represses photomorphogenesis by positively regulating phytochrome-interacting factors in the dark. The Plant cell. 2014; 26:3630-3645.
24. Yan Y, Li C, Dong X, Li H, Zhang D, Zhou Y, et al. MYB30 is a key negative regulator of *Arabidopsis* photomorphogenic development that promotes PIF4 and PIF5 protein accumulation in the light. The Plant cell. 2020; 32:2196-2215.
25. Oh J, Park E, Song K, Bae G, Choi G. PHYTOCHROME INTERACTING FACTOR8 inhibits phytochrome a-mediated far-red light responses in Arabidopsis. The Plant cell. 2020; 32: 186-205.
26. Oh E, Yamaguchi S, Kamiya Y, Bae G, Chung W, Choi G. Light activates the degradation of PIL5 protein to promote seed germination through gibberellin in *Arabidopsis*. The Plant journal. 2006; 47:124-139.
27. Wu G, Zhao Y, Shen R, Wang B, Xie Y, Ma X, et al. Characterization of maize phytochrome-interacting factors in light signaling and photomorphogenesis. Plant physiology. 2019; 181:789-803.
28. Liu X, Chen C, Wang K, Luo M, Tai R, Yuan L, et al. PHYTOCHROME INTERACTING FACTOR3 associates with the histone deacetylase HDA15 in repression of chlorophyll biosynthesis and photosynthesis in etiolated *Arabidopsis* seedlings. The Plant cell. 2013; 25:1258-1273.
29. Cheminant S, Wild M, Bouvier F, Pelletier S, Renou J, Erhardt M, et al. DELLAs regulate chlorophyll and carotenoid biosynthesis to prevent photooxidative damage during seedling deetiolation in Arabidopsis. The Plant cell. 2011; 23:1849-1860.
30. Song Y, Yang C, Gao S, Zhang W, Li L, Kuai B. Age-triggered and dark-induced leaf senescence require the bHLH transcription factors PIF3, 4, and 5. Molecular plant. 2014; 7:1776-1787.
31. Sharma S, Agarwal S.K. Plant leaf senescence: integrating multiple environmental and internal cues. Senescence Signalling and Control in Plants. 2019; Chapter 3: 29-40.
32. Kumar S, Lucyshyn D, Jaeger K, Alós E, Alvey E, Harberd N, et al. Transcription factor PIF4 controls the thermosensory activation of flowering. Nature. 2012; 484:242-245.
33. Thines, B, Youn, Y, Duarte, M, Harmon, F. The time of day effects of warm temperature on flowering time involve PIF4 and PIF5. Journal of experimental botany 2014, 65, 1141-1151.
34. Sellaro R, Pacín M, Casal J. Diurnal dependence of growth responses to shade in Arabidopsis: role of hormone, clock, and light signaling. Molecular plant. 2012; 5:619-628.
35. Ma D, Li X, Guo Y, Chu J, Fang S, Yan C, et al. Cryptochrome 1 interacts with PIF4 to regulate high temperature-mediated hypocotyl elongation in response to blue light. Proceedings of the National Academy of Sciences of the United States of America. 2016; 113:224-233.
36. Pedmale U, Huang S, Zander M, Cole B, Hetzel J, Ljung K, et al. Cryptochromes interact directly with PIFs to control plant growth in limiting blue light. Cell. 2016; 164:233-245.

37. Oh E, Kim J, Park E, Kim J, Kang C, Choi G. PIL5, a phytochrome-interacting basic helix-loop-helix protein, is a key negative regulator of seed germination in *Arabidopsis thaliana*. The Plant cell. 2004; 16:3045-3058.
38. Shin J, Kim K, Kang H, Zulfugarov I, Bae G, Lee C, et al. Phytochromes promote seedling light responses by inhibiting four negatively-acting phytochrome-interacting factors. Proceedings of the National Academy of Sciences of the United States of America. 2009; 106:7660-7665.
39. Xie C, Zhang G, An L, Chen X, Fang R. Phytochrome-interacting factor-like protein OsPIL15 integrates light and gravitropism to regulate tiller angle in rice. Planta. 2019; 250: 105-114.
40. Zhou J, Liu Q, Zhang F, Wang Y, Zhang S, Cheng H, et al. Overexpression of OsPIL15, a phytochrome-interacting factor-like protein gene, represses etiolated seedling growth in rice. Journal of integrative plant biology. 2014; 56:373-387.
41. Xiang S, Wu S, Zhang H, Mou M, Chen, Y, Li D, et al. The PIFs redundantly control plant defense response against Botrytis cinerea in *Arabidopsis*. Plants (Basel). 2020; 9:1246.
42. Proveniers M, van Zanten M. High temperature acclimation through PIF4 signaling. Trends in plant science. 2013; 18:59-64.
43. Balcerowicz M. PHYTOCHROME-INTERACTING FACTORS at the interface of light and temperature signalling. Physiologia plantarum 2020, 169, 347-356.
44. Wang F, Lian H, Kang C, Yang H. Phytochrome B is involved in mediating red light-induced stomatal opening in *Arabidopsis thaliana*. Molecular plant. 2010; 3: 246-259.
45. Xu D, Deng X. CBF-phyB-PIF module links light and low temperature signaling. Trends in plant science 2020; 25:952-954.
46. Jiang B, Shi Y, Zhang X, Xin X, Qi L, Guo H, et al. PIF3 is a negative regulator of the CBF pathway and freezing tolerance in *Arabidopsis*. Proceedings of the National Academy of Sciences of the United States of America. 2017;114:E6695-E6702.
47. Jiang B, Shi Y, Peng Y, Jia Y, Yan Y, Dong X, et al. Cold-induced CBF-PIF3 interaction enhances freezing tolerance by stabilizing the phyB thermosensor in Arabidopsis. Molecular plant. 2020; 13:894-906.
48. Zhao C, Zhang Z, Xie S, Si T, Li Y, Zhu J. Mutational evidence for the critical role of CBF transcription factors in cold acclimation in *Arabidopsis*. Plant physiology. 2016; 171:2744-2759.
49. Jia Y, Ding Y, Shi Y, Zhang X, Gong Z, Yang S. The *cbfs* triple mutants reveal the essential functions of CBFs in cold acclimation and allow the definition of CBF regulons in *Arabidopsis*. New phytologist. 2016; 212: 345-353.
50. Qiu J.R, Xiang X.Y, Wang J.T, Xu W.X, Huang Z. MfPIF1 of resurrection plant *myrothamnus flabellifolia* plays a positive regulatory role in responding to drought and salinity stresses in *Arabidopsis*. International Journal of Molecular Sciences. 2020; 21:3011.
51. Huai J, Zhang X, Li J, Ma T, Zha P, Jing Y, et al. SEUSS and PIF4 coordinately regulate light and temperature signaling pathways to control plant growth. Molecular plant. 2020; 13:1825.
52. Koini M, Alvey L, Allen T, Tilley C, Harberd N, Whitelam G, et al. High temperature-mediated adaptations in plant architecture require the bHLH transcription factor PIF4. Current biology. 2009; 19:408-413.
53. Fiorucci A, Galvão V, Ince Y, Boccaccini A, Goyal A, Allenbach Petrolati L, et al. PHYTOCHROME INTERACTING FACTOR 7 is important for early responses to elevated temperature in *Arabidopsis* seedlings. New phytologist. 2020; 226: 50-58.
54. Oh E, Kang H, Yamaguchi S, Park J, Lee D, Kamiya Y, Choi G. Genome-Wide Analysis of genes targeted by PHYTOCHROME INTERACTING FACTOR 3-LIKE5 during seed germination in *Arabidopsis*. The Plant Cell. 2009; 21: 403-419.
55. Kim J, Kang H, Park J, Kim W, Yoo J, Lee N, et al. PIF1-interacting transcription factors and their binding sequence elements determine the in vivo targeting sites of PIF1. The Plant cell. 2016; 28:1388-1405.
56. Gao Y, Wu M, Zhang M, Jiang W, Liang E, Zhang D, et al. Roles of a maize phytochrome-interacting factors

protein ZmPIF3 in regulation of drought stress responses by controlling stomatal closure in transgenic rice without yield penalty. Plant molecular biology. 2018; 97: 311-323.

57. Gao Y, Jiang W, Dai Y, Xiao N, Zhang C, Li H, et al. A maize phytochrome-interacting factor 3 improves drought and salt stress tolerance in rice. Plant molecular biology. 2015; 87:413-428.
58. Du L, Huang X, Ding L, Wang Z, Tang D, Chen B, et al. TaERF87 and TaAKS1 synergistically regulate TaP5CS1/TaP5CR1-mediated proline biosynthesis to enhance drought tolerance in wheat. New Phytologist 2023; 237:232-250.
59. Chou K.C, Shen H.B. Cell-PLoc 2.0: an improved package of web-servers for predicting subcellular localization of proteins in various organisms. Natural Science. 2010; 2:1090-1103.
60. Elisabeth G, Alexandre G, Christine H, Ivan I, Appel R. D, Amos B. ExPASy: the proteomics server for in-depth protein knowledge and analysis. Nucleic Acids Research. 2003; 31:3784-3788.
61. Larkin M, Blackshields G, Brown N, Chenna R, McGettigan P, McWilliam H, et al. Clustal W and Clustal X version 2.0. Bioinformatics (Oxford, England). 2007; 23:2947-2955.
62. Kumar S, Stecher G, Tamura K. MEGA7: Molecular Evolutionary Genetics Analysis Version 7.0 for Bigger Datasets. Molecular biology and evolution. 2016; 33:1870-1874.
63. Krzywinski M, Schein J, Birol I, Connors J, Gascoyne R, Horsman D, et al. Circos: an information aesthetic for comparative genomics. Genome research. 2009; 19:1639-1645.
64. Chen C, Chen H, Zhang Y, Thomas H.R, Xia R. TBtools: an integrative toolkit developed for interactive analyses of big biological data. Molecular Plant. 2020; 13:1194-1202.
65. Lescot M. PlantCARE, a database of plant *cis*-acting regulatory elements and a portal to tools for in silico analysis of promoter sequences. Nucleic Acids Research. 2002; 30:325-327.
66. Choulet F, Alberti A, Theil S, Glover N, Barbe V, Daron J, et al. Structural and functional partitioning of bread wheat chromosome 3B. Science (New York, N.Y.). 2014; 345:1249721.
67. Li Q, Zheng Q, Shen W, Cram D, Fowler D, Wei Y, et al. Understanding the biochemical basis of temperature-induced lipid pathway adjustments in plants. The Plant cell. 2015; 27:86-103.
68. Ma S, Wang M, Wu J, Guo W, Chen Y, Li G, et al. WheatOmics: A platform combining multiple omics data to accelerate functional genomics studies in wheat. Molecular plant. 2021; 14:1965-1968.
69. Amirbakhtiar N, Ismaili A, Ghaffari M.R, Firouzabadi, F.N, Shobbar, Z. S, Jain M. Transcriptome response of roots to salt stress in a salinity-tolerant bread wheat cultivar. PLoS ONE. 2019; 14:e0213305.
70. Liu Z, Xin M, Qin J, Peng H, Ni Z, Yao Y, et al. Temporal transcriptome profiling reveals expression partitioning of homeologous genes contributing to heat and drought acclimation in wheat (*Triticum aestivum* L.). BMC plant biology. 2015; 15:152.
71. Livak K.J, Schmittgen T.D. Analysis of relative gene expression data using real-time quantitative PCR. Methods. 2002; 25:402-408.
72. Yoo S, Cho Y, Sheen J. *Arabidopsis* mesophyll protoplasts: a versatile cell system for transient gene expression analysis. Nature protocols. 2007; 2:1565-1572.
73. Shiu S, Bleecker A. Expansion of the receptor-like kinase/Pelle gene family and receptor-like proteins in Arabidopsis. Plant physiology. 2003; 132:530-543.
74. Xu G, Guo C, Shan H, Kong H. Divergence of duplicate genes in exon-intron structure. Proceedings of the National Academy of Sciences of the United States of America. 2012; 109:1187-1192.
75. Butler J, Kadonaga J. The RNA polymerase II core promoter: a key component in the regulation of gene expression. Genes & development. 2002; 16:2583-2592.
76. Wang Z, Gerstein M, Snyder M. RNA-Seq: a revolutionary tool for transcriptomics, Nature Reviews Genetics. 2009; 10:57-63..
77. Favero D.S. Mechanisms regulating PIF transcription factor activity at the protein level. Physiol Plant. 2020; 169:325-335.

78. Leitch A, Leitch I. Genomic plasticity and the diversity of polyploid plants. Science (New York, N.Y.). 2008; 320:481-484.
79. Maher C, Stein L, Ware D. Evolution of *Arabidopsis* microRNA families through duplication events. Genome research. 2006; 16:510-519.
80. Todaka D, Nakashima K, Maruyama K, Kidokoro S, Osakabe Y, Ito Y, et al. Rice phytochrome-interacting factor-like protein OsPIL1 functions as a key regulator of internode elongation and induces a morphological response to drought stress. Proceedings of the National Academy of Sciences of the United States of America. 2012; 109:15947-15952.
81. Wu G, Zhao Y, Shen R, Wang B, Wang H. Characterization of maize phytochrome-interacting factors in light signaling and photomorphogenesis. Plant physiology. 2019; 181:789-803.
82. Rosado D, Trench B, Bianchetti R, Zuccarelli R, Rodrigues Alves, F Purgatto, et al. Downregulation of PHYTOCHROME-INTERACTING FACTOR 4 influences plant development and fruit production. Plant physiology. 2019; 181:1360-1370.
83. Verma V, Ravindran P, Kumar P. Plant hormone-mediated regulation of stress responses. BMC plant biology 2016; 16:86.
84. Gabriele S, Rizza A, Martone J, Circelli P, Costantino P, Vittorioso P. The Dof protein DAG1 mediates PIL5 activity on seed germination by negatively regulating GA biosynthetic gene *AtGA3ox1*. The Plant journal. 2010; 61:312-323.
85. Nozue K, Harmer S, Maloof J. Genomic analysis of circadian clock-, light-, and growth-correlated genes reveals PHYTOCHROME-INTERACTING FACTOR5 as a modulator of auxin signaling in *Arabidopsis*. Plant physiology. 2011;156:357-372.
86. Hu W, Huang C, Deng X, Zhou S, Chen L, Li Y, et al. TaASR1, a transcription factor gene in wheat, confers drought stress tolerance in transgenic tobacco. Plant, cell & environment. 2013; 36:1449-1464.
87. Mei F, Chen B, Du L, Li S, Zhu D, Chen N, et al. A gain-of-function allele of a DREB transcription factor gene ameliorates drought tolerance in wheat. The Plant Cell. 2022; 34:4472-4494.
88. Mao H, Li S, Chen B, Jian C, Mei F, Zhang Y, et al. Variation in cis-regulation of a NAC transcription factor contributes to drought tolerance in wheat. Molecular Plant. 2022;15: 276-292.
89. Bi C, Yu Y, Dong C, Yang Y, Zhai Y, Du F, et al. The bZIP transcription factor TabZIP15 improves salt stress tolerance in wheat. Plant Biotechnology Journal. 2021;19: 209-211.
90. Sun H, Xie Y, Yang W, Lv Q, Chen L, Li J, et al. Membrane-bound transcription factor TaNTL1 positively regulates drought stress tolerance in transgenic *Arabidopsis*. Plant Physiology Biochemistry. 2022; 182:182-193.
91. Vlot AC, Dempsey DA, Klessig DF. Salicylic Acid, a multifaceted hormone to combat disease. Annual Review Of Phytopathology. 2009; 47:177-206.
92. Yang YX, Ahammed GJ, Wu C, Fan SY, Zhou YH. Crosstalk among jasmonate, salicylate and ethylene signaling pathways in plant disease and immune responses. Current Protein & Peptide Science. 2015; 16:450-461.
93. Cox KL. Stronger together: ethylene, jasmonic acid, and MAPK signaling pathways synergistically induce camalexin synthesis for plant disease resistance. The Plant Cell. 2022; 34:2829-2830.
94. Shi Q, Zhang H, Song X, Jiang Y, Liang R, Li G. Functional characterization of the maize phytochrome-interacting factors PIF4 and PIF5. Frontiers In Plant Science. 2018; 18:2273.
95. Shi H, Wang X, Mo X, Tang C, Zhong S, Deng XW. Arabidopsis DET1 degrades HFR1 but stabilizes PIF1 to precisely regulate seed germination. Proceedings of the National Academy of Sciences of the United States of America. 2015; 112: 3817-3822.
96. Saini K, Dwivedi A, Ranjan A. High temperature restricts cell division and leaf size by coordination of PIF4 and TCP4 transcription factors. Plant Physiology. 2022; 190:2380-2397.

97. Lau OS, Song Z, Zhou Z, Davies KA, Chang J, Yang X, et al. Direct control of SPEECHLESS by PIF4 in the high-temperature response of stomatal development. Current Biology. 2018; 28:1273-1280.
98. He Y, Li Y, Cui L, Xie L, Zheng C, Zhou G, et al. Phytochrome B negatively affects cold tolerance by regulating OsDREB1 gene expression through Phytochrome Interacting Factor-Like Protein OsPIL16 in Rice. Frontiers Plant Science. 2016; 26:1963.
99. Jeong J, Choi G. Phytochrome-interacting factors have both shared and distinct biological roles. Molecules and Cells. 2013; 35:371–380.
100. Wang W, Shinwari KI, Zhang H, Zhang H, Dong L, He F, et al. The bHLH transcription factor OsbHLH057 regulates iron homeostasis in rice. International Journal Of Molecular Sciences. 2022; 23:14869.
101. Huang Z, Tang R, Yi X, Xu W, Zhu P, Jiang C. Overexpressing Phytochrome Interacting Factor 8 of Myrothamnus flabellifolia enhanced drought and salt tolerance in *Arabidopsis*. International Journal Of Molecular Sciences. 2022; 23:8155.
102. Qiu JR, Xiang XY, Wang JT, Xu WX, Chen J, Xiao Y, et al. MfPIF1 of resurrection plant myrothamnus flabellifolia plays a positive regulatory role in responding to drought and salinity stresses in Arabidopsis. International Journal Of Molecular Sciences. 2020; 21:3011.
103. Nie N, Huo J, Sun S, Zuo Z, Chen Y, Liu Q, et al. Genome-wide characterization of the PIFs family in sweet potato and functional identification of IbPIF3.1 under drought and fusarium wilt stresses. International Journal Of Molecular Sciences. 2023; 24:4092.
104. Liu Z, Guo C, Wu R, Hu Y, Zhou Y, Wang J, et al. FLS2-RBOHD-PIF4 module regulates plant response to drought and salt stress. International Journal Of Molecular Sciences. 2022; 23:1080.
105. Zhang X, Huai J, Shang F, Xu G, Tang W, Jing Y, et al. A PIF1/PIF3-HY5-BBX23 transcription factor cascade affects photomorphogenesis. Plant Physiology. 2017; 174:2487-2500.
106. Wei K, Chen H. Comparative functional genomics analysis of bHLH gene family in rice, maize and wheat. BMC Plant Biology. 2018; 18:309.

Table 1 Characterization of PIFs in wheat.

Gene name	Locus_ID	Chr.	Position		Deduced polypeptide			instability index	GRAVY	Subcellular localization
			Start	End	Length (aa)	MW (KDa)	pI			
TaPIF1-1A	*TraesCS1A02G083000*	1A	66174370	66178373	507	54.41	6.31	79.06	-0.53	nucleus
TaPIF1-1B	*TraesCS1B02G100400*	1B	109210350	109213975	390	42.14	6.53	83.48	-0.74	nucleus
TaPIF1-1D	*TraesCS1D02G084200*	1D	67860728	67864379	502	53.89	6.37	75.51	-0.50	nucleus
TaPIF2-1A	*TraesCS1A02G212700*	1A	375470020	375478638	455	48.42	5.88	55.07	-0.47	nucleus
TaPIF2-1B	*TraesCS1B02G226200*	1B	406037910	406052114	454	48.37	6.61	56.43	-0.51	nucleus
TaPIF2-1D	*TraesCS1D02G215600*	1D	300860435	300872558	454	48.18	5.87	53.56	-0.46	nucleus
TaPIF3-2A	*TraesCS2A02G253900*	2A	386292343	386297900	347	36.86	6.05	69.09	-0.56	nucleus
TaPIF3-2B	*TraesCS2B02G273500*	2B	374801132	374803415	358	38.31	5.77	67.10	-0.59	nucleus
TaPIF3-2D	*TraesCS2D02G254400*	2D	306528220	306533600	350	37.46	5.79	64.95	-0.57	nucleus
TaPIF4.1-2A	*TraesCS2A02G461700*	2A	707567126	707569581	186	21.48	9.10	84.21	-0.83	nucleus

TaPIF4.1-2B	*TraesCS2B02G483300*	2B	680597075	680599355	188	21.72	9.10	82.57	-0.81	nucleus
TaPIF4.1-2D	*TraesCS2D02G461700*	2D	568090552	568092978	184	21.20	9.10	84.07	-0.79	nucleus
TaPIF4.2-2D	*TraesCS2D02G461900*	2D	568105788	568107153	157	18.41	8.64	88.02	-0.75	nucleus
TaPIF5-5A	*TraesCS5A02G049600*	5A	45369032	45372490	336	36.60	5.37	63.31	-0.63	nucleus
TaPIF5-5B	*TraesCS5B02G054800*	5B	59925570	59928669	338	36.87	6.61	66.58	-0.52	nucleus
TaPIF5-5D	*TraesCS5D02G060300*	5D	56950525	56953479	350	37.96	5.87	62.38	-0.505	nucleus
TaPIF6-5A	*TraesCS5A02G225700*	5A	441475674	441480524	458	46.14	6.26	57.98	-0.33	nucleus
TaPIF6-5B	*TraesCS5B02G224200*	5B	399297111	399301709	465	47.04	6.30	59.24	-0.31	nucleus
TaPIF6-5D	*TraesCS5D02G233000*	5D	340509122	340513309	468	47.15	6.17	57.67	-0.30	nucleus
TaPIF7-5A	*TraesCS5A02G376500*	5A	574193926	574200864	487	51.69	6.39	65.95	-0.59	nucleus
TaPIF7-5B	*TraesCS5B02G380200*	5B	558117488	558124468	447	47.69	6.08	61.79	-0.58	nucleus
TaPIF7-5D	*TraesCS5D02G386500*	5D	456338580	456345166	516	55.09	6.40	64.87	-0.58	nucleus
TaPIF8-5A	*TraesCS5A02G420200*	5A	606892572	606895950	420	44.61	6.53	70.81	-0.55	nucleus
TaPIF8-5B	*TraesCS5B02G422000*	5B	597217607	597223578	410	43.24	6.50	71.11	-0.57	nucleus
TaPIF8-5D	*TraesCS5D02G428400*	5D	486165825	486171802	414	43.56	6.05	71.26	-0.58	nucleus

TaPIF9-6A	*TraesCS6A02G358400*	6A	588454642	588459868	333	33.94	5.04	57.00	-0.444	nucleus
TaPIF9-6B	*TraesCS6B02G391100*	6B	665507149	665512393	360	37.03	5.74	58.24	-0.50	nucleus
TaPIF9-6D	*TraesCS6D02G341200*	6D	440362746	440368142	359	36.66	5.54	58.43	-0.46	nucleus
TaPIF10-6A	*TraesCS6A02G373500*	6A	596746992	596751431	302	33.58	7.25	66.55	-0.66	nucleus
TaPIF10-6B	*TraesCS6B02G411300*	6B	685198642	685202790	303	33.58	7.26	69.94	-0.71	nucleus
TaPIF10-6D	*TraesCS6D02G357700*	6D	451108081	451112377	297	33.01	7.83	66.59	-0.73	nucleus
TaPIF11-7A	*TraesCS7A02G126900*	7A	81631856	81636020	331	36.21	6.54	84.61	-0.81	nucleus
TaPIF11-7B	*TraesCS7B02G026300*	7B	25586884	25591260	339	36.71	6.51	85.37	-0.75	nucleus
TaPIF11-7D	*TraesCS7D02G124700*	7D	78189000	78193774	338	36.65	6.68	86.66	-0.79	nucleus

Figure legends

Fig 1. Phylogenetic analysis of PIF families in *Arabidopsis thaliana*, rice, maize, *Brachypodium distachyon*, and wheat. (A) Relationships between PIFs in wheat, *Oryza sativa*, *Arabidopsis*, *B. distachyon*, and *Zea mays*. The neighbor-joining phylogenetic tree was constructed using the full-length protein sequences of all known PIFs in each species. The proteins could be classified into five groups (I–V). The bootstrap scores shown at each node were calculated from 1000 replicates. (B) Distribution of PIFs among each group in each of the five species.

Fig 2. *PIF* synteny analysis. (A) Synteny analysis of *PIFs* in the wheat genome. Isomorphic *TaPIF* gene pairs are connected with red lines. (B) Synteny analysis of *Di19* genes in wheat, *Oryza sativa*, *Arabidopsis thaliana*, and *Brachypodium distachyon*. Gray lines indicate collinearity between the wheat genome and those of other plant species. Red lines show isomorphic *PIF* gene pairs.

Fig 3. Phylogenetic relationships, conserved motifs, and gene structures of TaPIFs. Phylogenetic analysis of PIFs in wheat and four other plant species clustered the proteins into five groups. Left, conserved motifs among the TaPIFs. Right, gene structures of *TaPIF*s. Untranslated regions are indicated with green boxes, exons are indicated with yellow boxes, and introns are represented by black lines.

Fig 4. Analysis of *TaPIF* promoter *cis*-elements. Analysis of *TaPIF* promoter *cis*--elements. The 2-kb promoter sequences of *TaPIF*s were analyzed to identify predicted *cis*-elements. The identified *cis*-elements were classified into three

categories: hormone-related, development-related, and abiotic stress-related. The color intensity and number in each square indicate the number of each type of *cis*-element in the promoter region of the indicated gene. The distribution of *cis*-elements in each category for each gene is shown at right.

Fig 5. Tissue-specific *TaPIF* expression patterns. Expression profiles of *TaPIF* genes across multiple tissue types. Analyses were conducted using publicly-available RNA-seq data. R, S, and L indicate the root, stem, and leaf tissue, respectively, from seedlings at the five-leaf stage. FL refers to the flag leaf of wheat plants at the booting and heading stages. YS5 and YS15 indicate young spikes from wheat plants at the booting and heading stages, respectively. GR5, GR10, GR15, and GR20 refer to grains at 5, 10, 15, and 20 d post-fertilization, respectively. All expression values were log_2 transformed.

Fig 6. *TaPIF* expression in response to biotic stress. Expression profiles of *TaPIF* genes in wheat after infection with powdery mildew or stripe rust. Analyses were conducted using publicly-available RNA-seq data. All expression values were log_2 transformed.

Fig 7. Gene expression patterns of *TaPIF*s in response to abiotic stress or phytohormone treatment. (A) Expression profiles of *TaPIF* genes in wheat exposed to drought (D), heat (H), or combined drought and heat (D&H). (B) Expression profiles of *TaPIF* genes in wheat exposed to salt or cold stress. CK1 and CK2 were the control samples for the salt and cold stress treatments, respectively. (C)

Expression profiles of *TaPIF* genes in wheat exposed to abscisic acid (ABA), 6-benzylaminopurine (6-BA), salicylic acid (SA), gibberellic acid (GA), or jasmonic acid (JA). CK1 was the control for the ABA, 6-BA, and SA treatments; CK2 was the control for the GA and JA treatments. Analyses were conducted using publicly-available RNA-seq data. All expression values were log_2 transformed.

Fig 8. Expression profiles of *TaPIFs* treated with polyethylene glycol (PEG) or abscisic acid (ABA). *TaPIF* expression in wheat shoots and roots in response to treatment with 20% PEG-6000 or 100 μM ABA. Expression was measured with quantitative reverse transcription PCR. Data are presented as the mean ± standard deviation (n = 3).

Fig 9. Subcellular localization and transactivation activity of TaPIFs in wheat. (A) Transactivation activity analyses of five TaPIFs in yeast. The tested proteins were TaPIF2, TaPIF5, and TaPIF10. Transformed yeast cells were grown on the synthetic media SD/-Trp, SD/-Trp/-His/-Ade, and SD/-Trp/-His/-Ade/x-α-gal. (B) Subcellular localization of TaPIFs in wheat mesophyll protoplasts. Protoplasts were transfected with 35S::*TaPIF2-GFP*, 35S::*TaPIF5-GFP*, 35S::*TaPIF10-GFP*, or the 35S::*GFP* control and visualized with laser scanning confocal microscopy. Scale bar = 30 μm.

Supplemental Information

Fig S1. Conserved motifs in TaPIF proteins. Conserved motifs were identified with the MEME suite of tools and visualized with TBtools.

Table S1. Basic information about *PIF*s in wheat, *Arabidopsis*, rice, maize, and *Brachypodium distachyon*.

Table S2. The *Ka*/*Ks* ratios and duplication times of paralogous *TaPIF*s in wheat, rice, *Arabidopsis*, maize, and *Brachypodium distachyon*.

Table S3. Conserved motifs in TaPIF proteins.

Table S4. *Cis*-regulatory elements in *TaPIF* promoters.

Table S5. *TaPIF* expression levels in fragments per kilobase of transcript per million mapped reads (FPKM).

Table S6. Primers used in this study.

Fig 1 Click here to access/download;Figure;Fig 1.tif

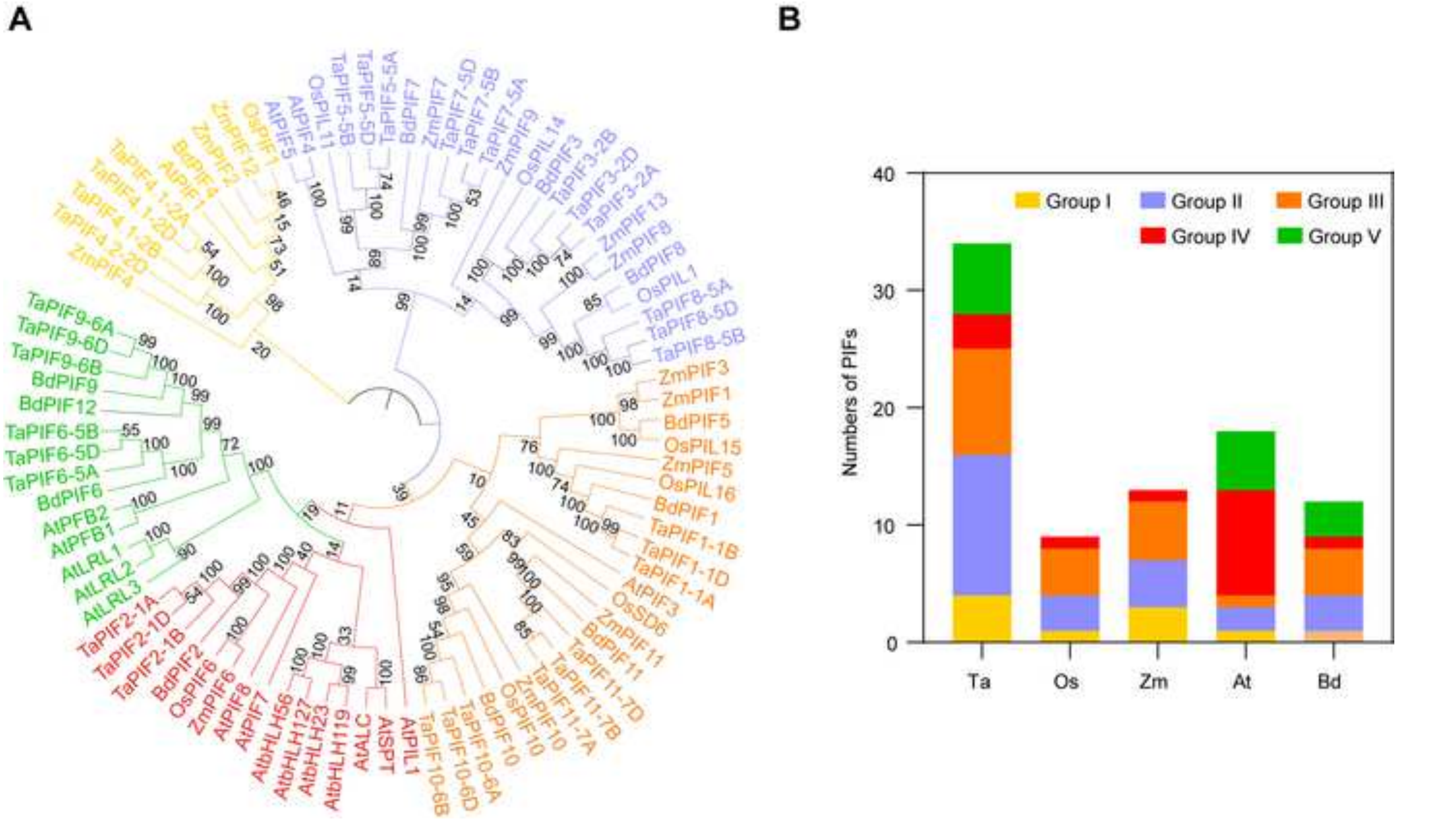

Fig 2

Click here to access/download;Figure;Fig 2.tif

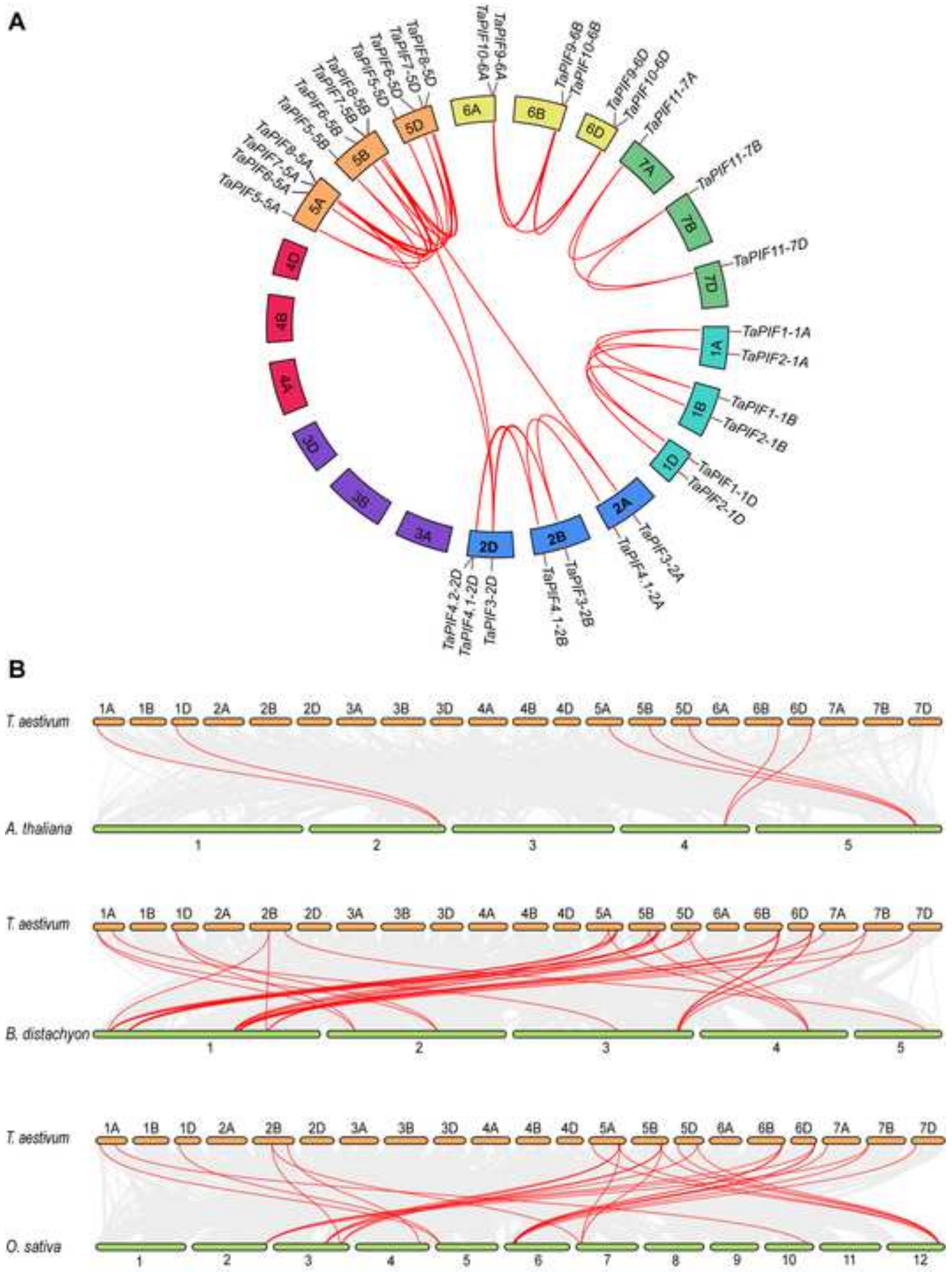

Fig 3

Click here to access/download;Figure;Fig 3.tif

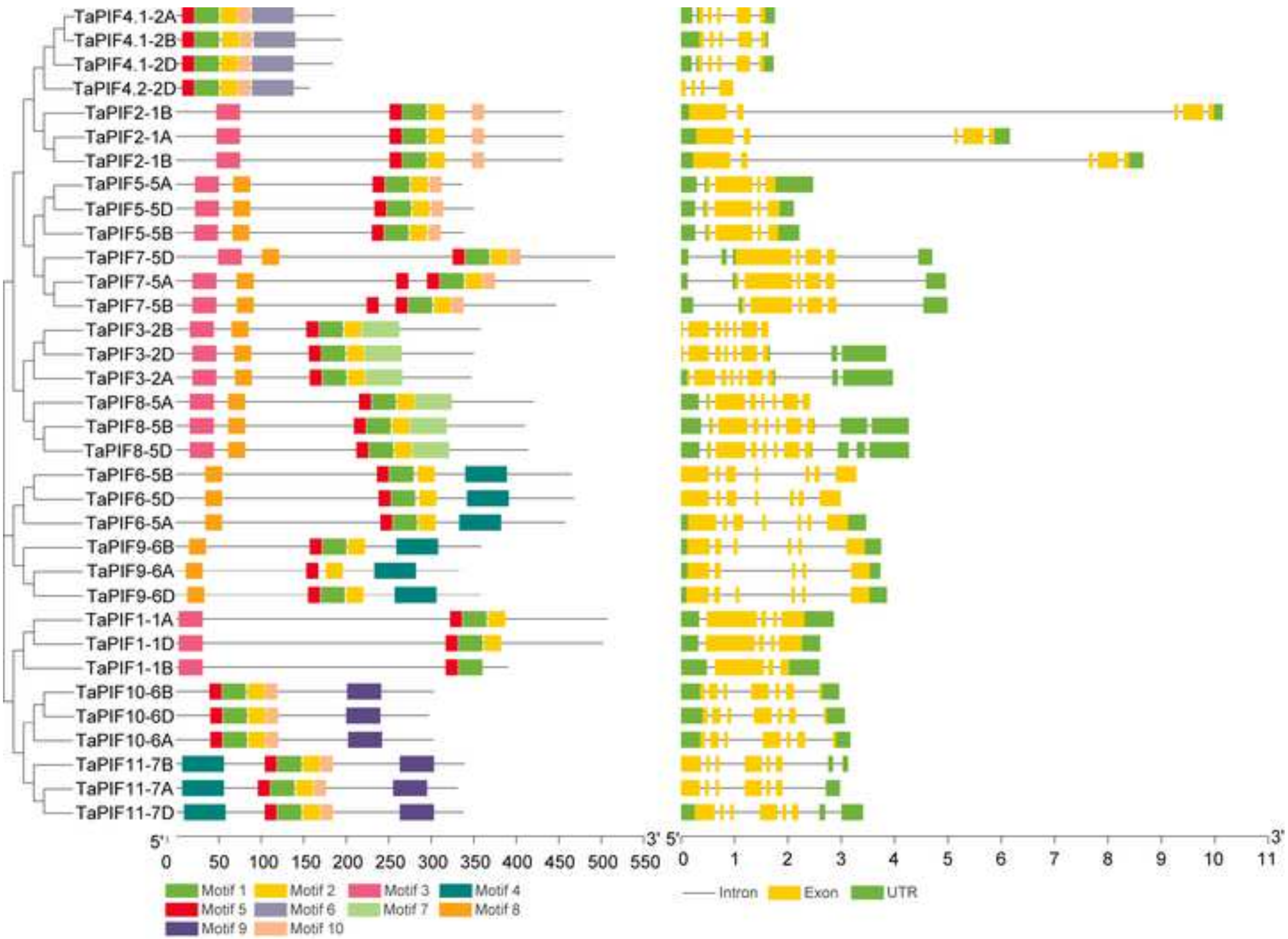

Fig 4

Click here to access/download;Figure;Fig 4.tif

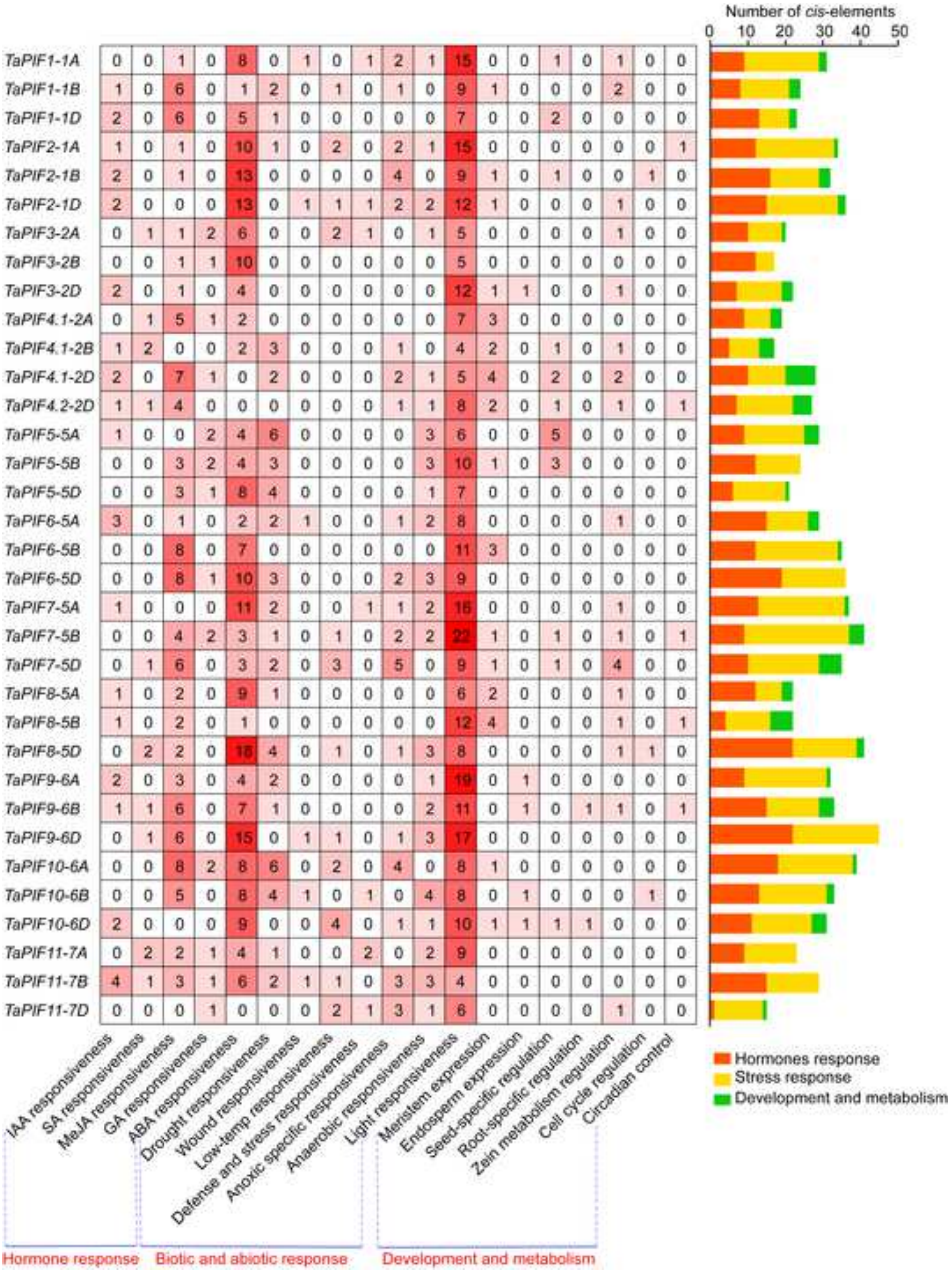

Fig 5

Click here to access/download;Figure;Fig 5.tif

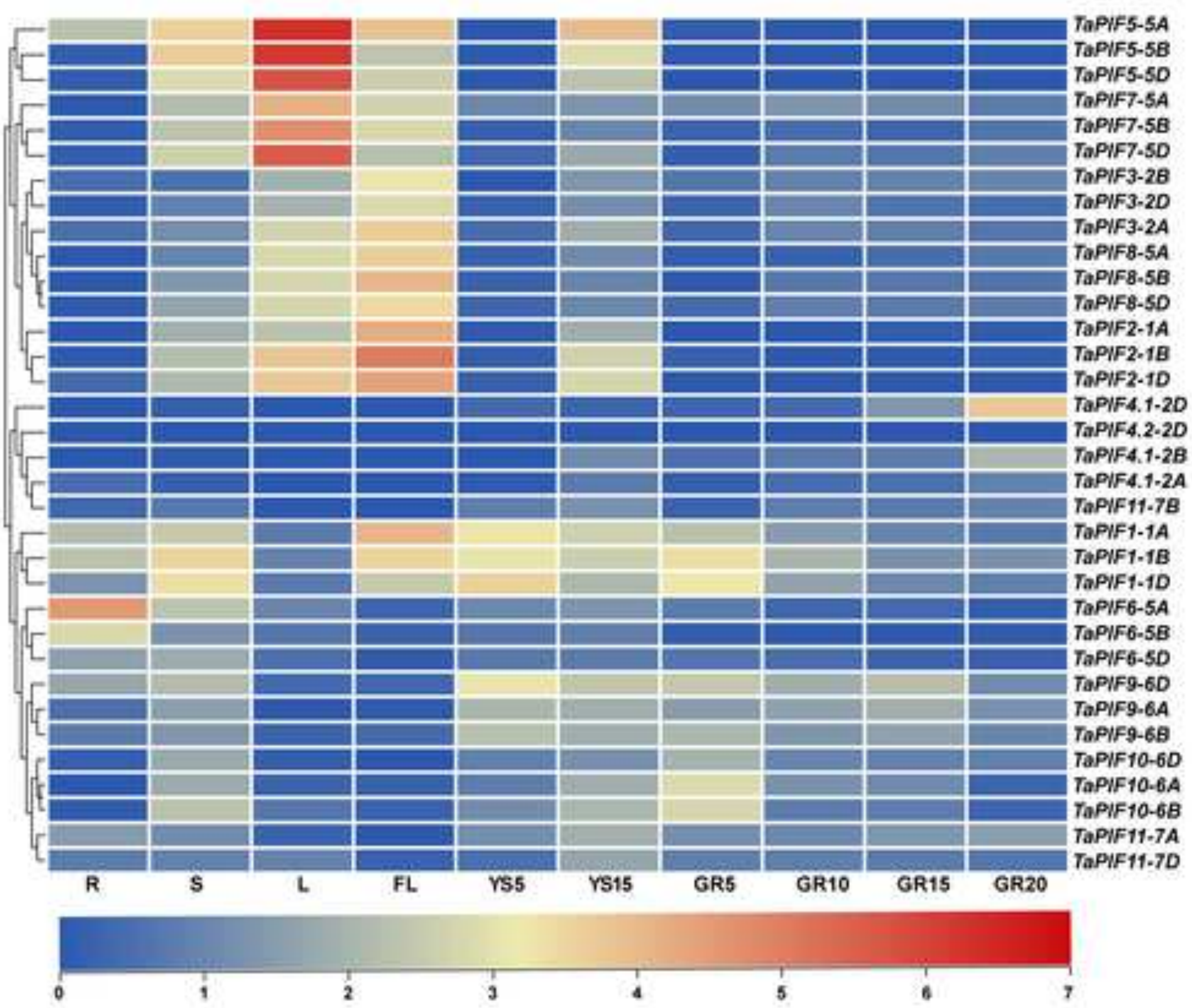

 Click here to access/download;Figure;Fig 6.tif

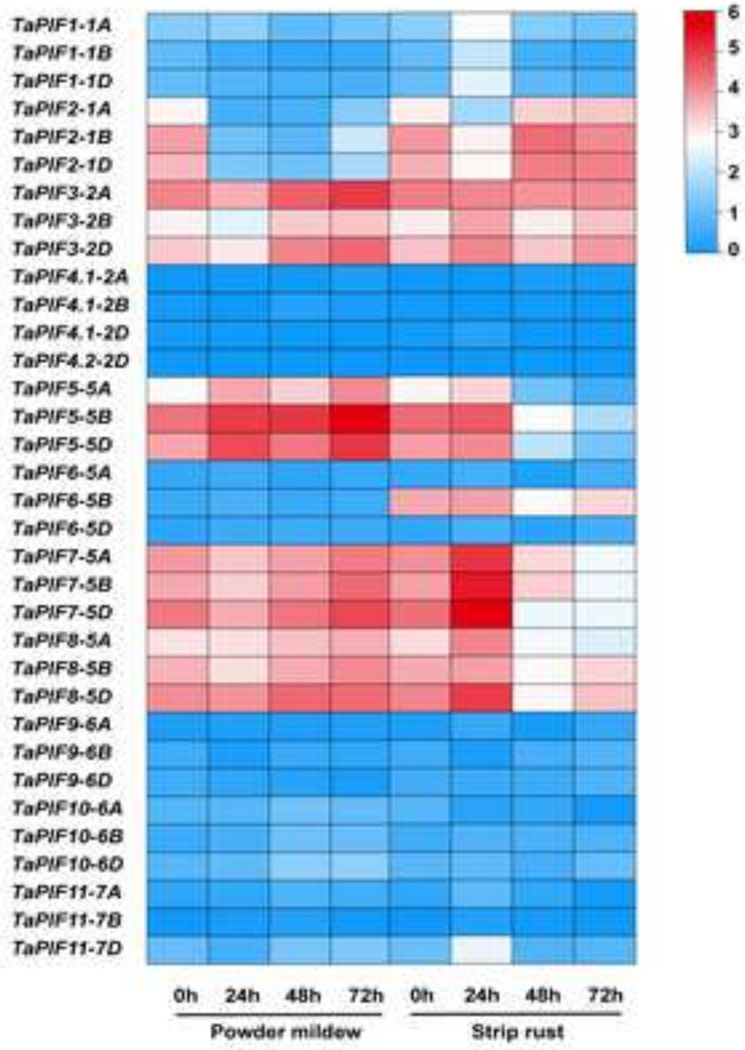

Click here to access/download;Figure;Fig 7.tif

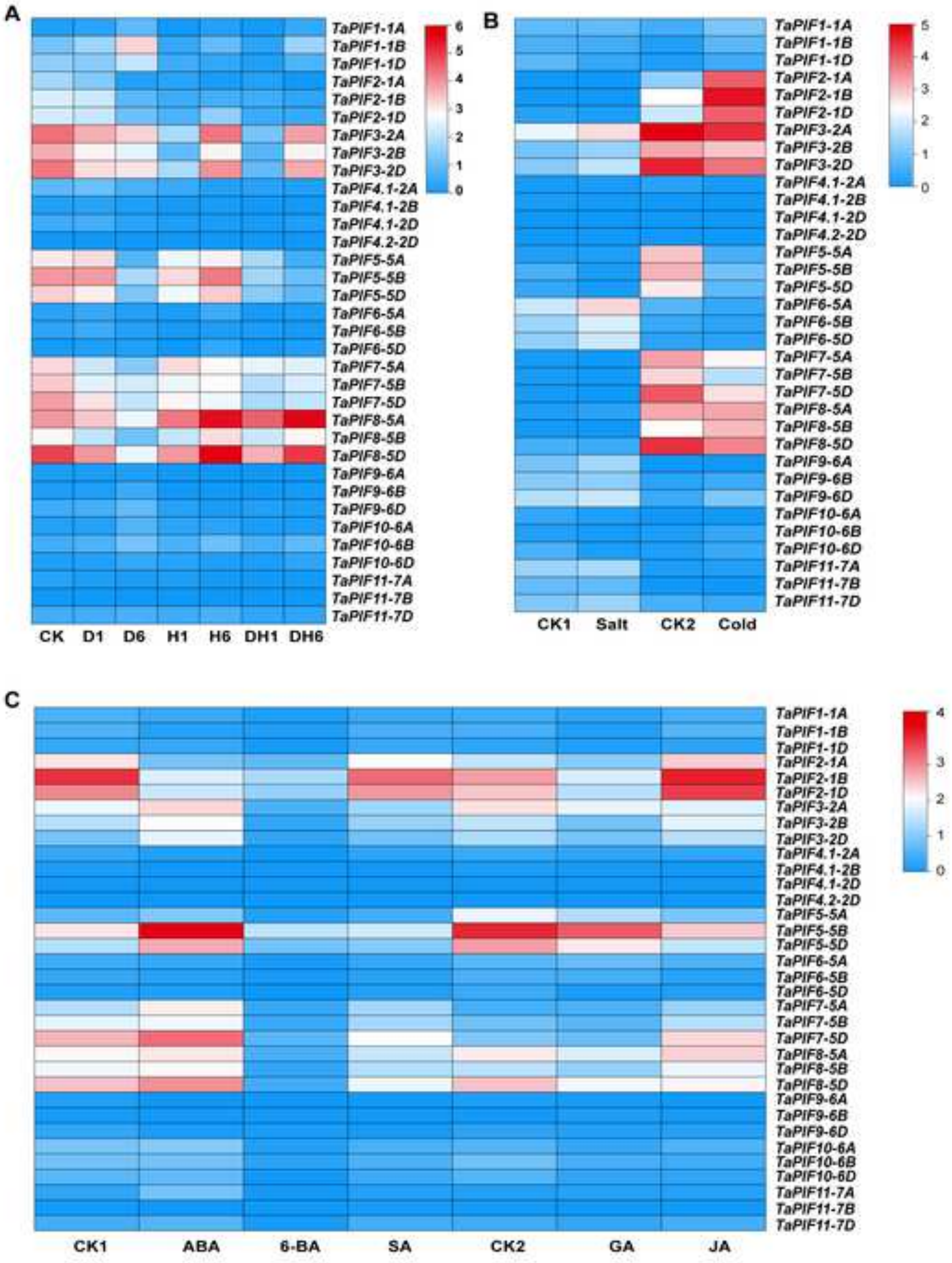

Fig 8

Click here to access/download;Figure;Fig 8.tif

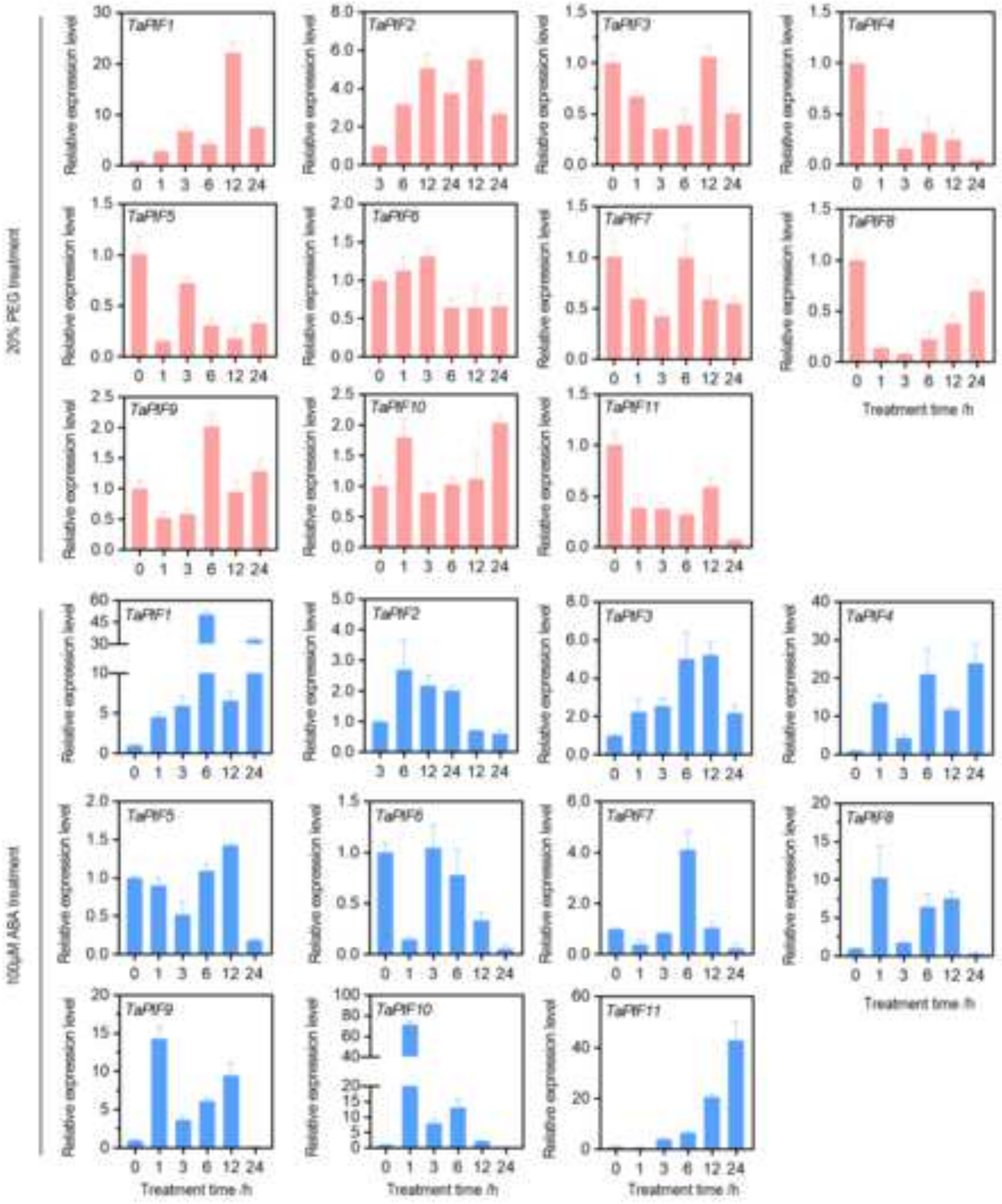

Click here to access/download;Figure;Figure 9.tif

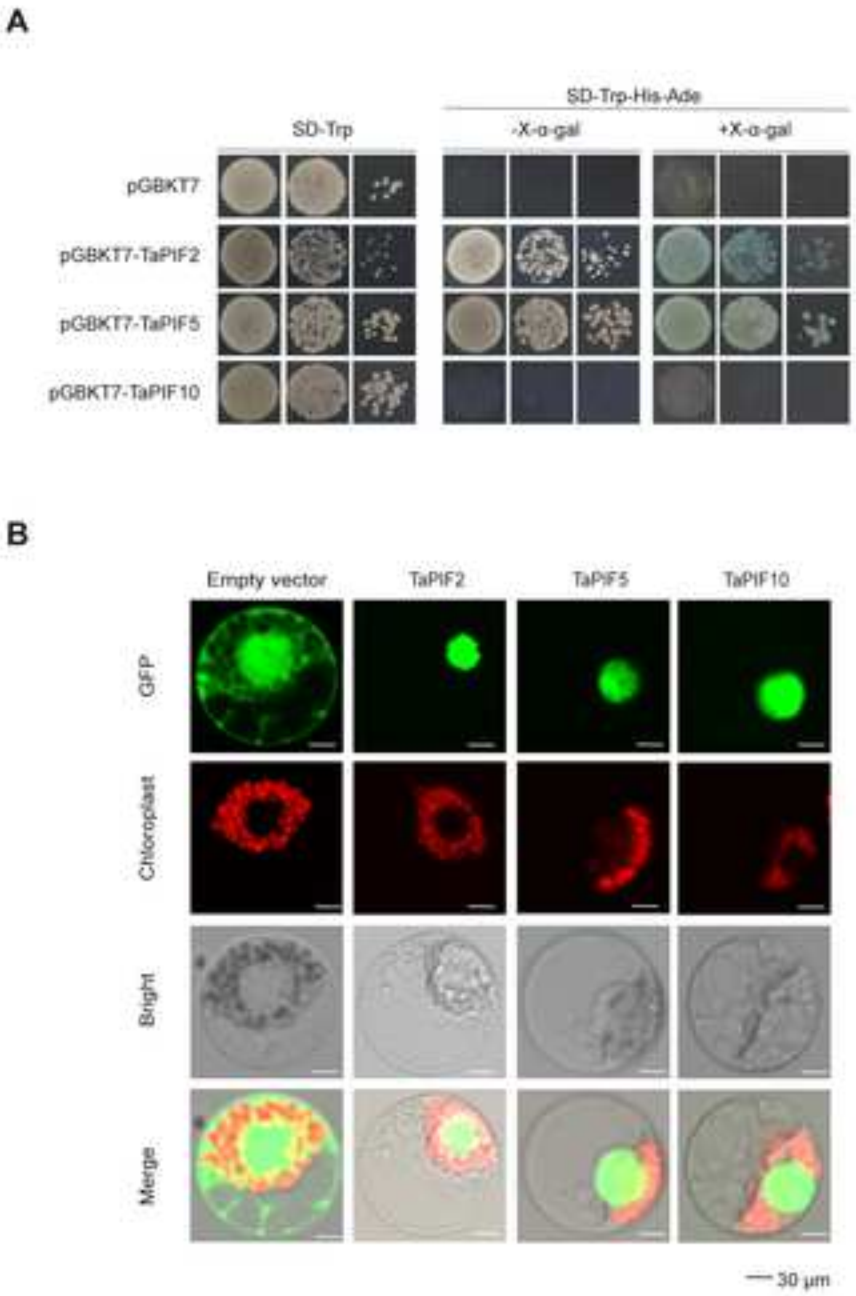

Fig S1

Click here to access/download
Supporting Information
Fig S1.tif

Table S1

Click here to access/download
Supporting Information
Table S1.xlsx

Table S2

Click here to access/download
Supporting Information
Table S2.xlsx

Table S3

Click here to access/download
Supporting Information
Table S3.xlsx

Table S4

Click here to access/download
Supporting Information
Table S4.xlsx

Table S5

Click here to access/download
Supporting Information
Table S5.xlsx

Table S6

Click here to access/download
Supporting Information
Table S6 .xlsx

Table 1

Click here to access/download
Supporting Information
Table 1 TaPIF information.xlsx
