# Supplementary material for: Genome-wide identification and comprehensive analysis of the phytochrome-interacting factor (PIF) gene family in wheat
Source: PLoS One. 2024 Jan 5;19(1):e0296269. doi: 10.1371/journal.pone.0296269 (PMC10769075; doi:10.1371/journal.pone.0296269)
Supplement: Attachment — Submitted filename: Response to Reviewers.doc [file pone.0296269.s011.doc]

#### **Reply to the editors and reviewers**

#### Reference: PONE-D-23-27525

Title: Genome-wide identification and comprehensive analysis of the phytochrome-interacting factor (*PIF*) gene family in wheat

Authors: Hua Zhuang, Zhen Guo, Jian Wang, Tianqing Chen

Dear editors,

We thank you for your timely response to our submission and offer our thanks to the reviewers for their highly constructive, thoughtful comments and suggestions regarding our study. We have revised our article to avoid overlapping text and previous research, including introduction, conclusion, and other sections. We have revised the manuscript as appropriate based on their suggestions and have done our best to address all concerns raised by each reviewer. Enclosed, please find our point-by-point responses. We are very grateful for the editorial and scientific guidance provided in this review process, which has greatly improved the quality of our study and will inform our research going forward. We thank you kindly again for your consideration. Please let us know if any further information is needed.

**Response to Reviewer #1**

The manuscript of Zhuang et al. presents the identification about the roles and expression patterns of phytochrome interacting factor (PIF) gene family in wheat. The authors systematically analyzed the functions of TaPIFs by bioinformatics, expression patterns, subcellular localization and transcriptional activation. Overall, the evolutionary and bioinformatics analyses are extensive, well presented, supported by highquality and well-organized figures. Some revisions are however necessary to add. The manuscript is also in need of English language and grammar revisions that I will not report here, due to a too long list of indications.

Response: Thank you for taking the time to review our manuscript. We appreciate your valuable feedback and suggestions, which have greatly contributed to improving the quality of our work. We have carefully considered each of your comments and made the necessary revisions accordingly. We have also sent the revised manuscript to a professional manuscript editing company staffed by American PhDs trained in the life sciences for correction and polishing of our English.

**Response to Reviewer #2**

This study by Zhuang et al. examined the PIF gene family in wheat using a variety of sequencebased analyses. The authors presented multiple results including phylogenetic analysis, gene and protein motif analysis, promoter *cis*-regulatory element comparison, gene expression data, and even subcellular localization results to support their study. In general, the findings have the potential to be a useful foundation for further studies involving TaPIFs.

Despite the comprehensive analysis by the authors, there are several critical questions that were not addressed in this version of the manuscript. Importantly, the authors need to show that the 34 TaPIFs are true PIF homologs. Arabidopsis PIFs are well-studied and contain several key features including a conserved bHLH domain as well as APB/APA domains. Furthermore, sequence alignment against other PIF homologs were not shown in this version of the manuscript, making it difficult to assess the accuracy of the identification. Moreover, the authors need to clarify why their phylogenetic analysis showed *O. sativa*, *Z. mays*, and *Arabidopsis* to have larger number of PIFs than what is currently known in literature.

Response: We thank the reviewer for their constructive comments and useful suggestions, the latter of which have helped us improve the manuscript. We re-identified TaPIF proteins in wheat according to the reviewer's suggestion, which were confirmed to contain conserved bHLH domain and APB domain one by one, and confirmed that a total of 18 TaPIF protein sequences belonged to 6 homeologous groups distributed on the A, B, and D subgenomes (see Page 5, Lines 86-88 and lines 89-91). Multiple sequences were compared for 44 PIF proteins from wheat, rice, *Arabidopsis*, *Brachysteta dipaniculata*, and maize to enhance the reliability of the results, and typical conserved domains were identified (Figure S1). In addition, we also corrected the PIF protein in *Arabidopsis*, rice, and maize, and reconstructed the phylogenetic tree (Figure 1A), and the corrected PIF protein number in each species was consistent with the references.

“A total of 18 TaPIF proteins with conserved domains of bHLH and APB were identified (Table 1, Figure S1).”

“The similarity percentage of TaPIF proteins were 96.4% (TaPIF1s), 97.1% (TaPIF2s), 94.8% (TaPIF3s), 90.4% (TaPIF4s), 91.7% (TaPIF5s), 93.1% (TaPIF6s), respectively.”

Below are some major issues that need to be addressed:

(1) While the authors identified 34 TaPIFs via sequence similarity to known AtPIFs, the sequence alignment or similarity percentage/score were not provided in the manuscript. Hence, it is not possible to assess whether the identified proteins are truly homologs.

Response: We appreciate the reviewer's suggestions. We have revised the section by providing sequence alignment in the manuscript, see Figure S1 .

(2) The provision of instability indexes, GRAVY scores, and predicted subcellular localization is good information. To highlight the significance of these results, the authors can compare these indexes against all AtPIFs, which are well-studied.

Response: We appreciate the reviewer's suggestions. The physicochemical properties of PIF protein in *Arabidopsis*, rice, *Brachypodium distachyon* and maize were added (see Table S1), and compare instability indexes, GRAVY scores, and predicted subcellular localization against TaPIFs for highlight the significance of these results, see Page 5, lines 99-103.

“In addition, we also predicted the above parameters of PIF protein in rice, Arabidopsis, Brachypodium distachyon, and maize (Table S1), finding these proteins were similar to TaPIF proteins on instability indexes, GRAVY scores, and subcellular localization. In particular, the hydrophilicity of PIF protein in Arabidopsis (-0.95 to 0.562) were generally higher than that in wheat.”

(3) It is unclear how the authors conducted the phylogenetic analysis. In the introduction, the authors described *O. sativa* having only seven PIFs, *Z. mays* has eight PIFs, while *Arabidopsis* has nine PIFs. However, in Figure 1 and Table S1, these plant species were shown to have more PIFs than previously described. In Table S1, genes such as SPT, ALC, etc. were listed as a PIF gene. This is not true.

Response: We appreciate the reviewer's suggestions. We mistakenly included members of all 15 subgroups of the bHLH superfamily when in fact there were members that did not belong to the PIF protein. We carefully conducted amino acid multiple sequence alignment and reconstructed the developmental tree, and excluded the members of the bHLH domain and APB domain that did not have the typical conserved PIF, and revised in Figure 1 and Table S1. At the same time, the reference in the preface is corrected to make the preceding.

(4) The identification of common motifs amongst TaPIFs is compelling evidence to support the phylogeny analysis. To improve this data set, the authors should identify the presence of active phytochrome B-binding (APB) and active phytochrome A-binding (APA) motifs in the TaPIFs. APAs and APBs are defining features of PIFs, in addition to having conserved bHLH domains.

Response: We appreciate the reviewer's suggestions. We carefully performed multiple amino acid alignment and mapped the typically conserved bHLH and APB domains of PIF on the TaPIF amino acid sequence of wheat, See Figure S1.

(5) The authors mentioned that motifs 1, 2, and 5 have the same arrangement in all TaPIFs, but did not further describe what these motifs contain and their significance. Is this the bHLH domain? If so, how do these domains compare with AtPIFs?

Response: We appreciate the reviewer's suggestions. We describe what these motifs contain in the manuscript, see Page 7, lines 145-146.

“All of the TaPIFs contained the same arrangement of motif 2 and motif 5*,* which represent bHLH and APB domains, the features of PIF proteins, respectively.”

(6) Figures 6 and 7: It will be helpful to link the findings here to Figure 4 (*cis*-elements in promoter). This can elucidate if the *cis*-elements identified in Figure 4 result in the biotic and abiotic responses seen in Figures 6 and 7.

Response: We are grateful for the reviewer's suggestion. We analyzed and added the relationship between *cis*-elements identified in Figure 4 and the result in the biotic and abiotic responses seen in Figures 6 and 7 according to the reviewer's suggestions, see Page 10, lines 203-207; lines 212-215, and Page 10, lines 219-222 and Page 11, lines 226-235.

“Combined with the above *cis*-acting element identification, the promoter regions of *TaPIF1-1A/B/D*, *TaPIF2-1A/B/D*, *TaPIF5-5A/B/D*, and *TaPIF6-5A/B/D* identified defense and stress responses, SA response, or MeJA response elements (Figure 4, Table S5), which may be the reason for the response of these genes to the infection of stripe rust or powdery mildew.”

“Under drought conditions, *TaPIF1-1A/B/D* were up-regulated compared with control plants; there were no significant changes in *TaPIF4* expression levels, and the remainder of *TaPIF* genes were down-regulated (Figure 7A, Table S6), which consistent with their promoter region containing drought-response elements like MBS or DRE (Figure 4).”

“Besides, *TaPIF1-1A/B/D*, *TaPIF2-1A/B/D*, and *TaPIF6-5D* were up-regulated under cold conditions, whereas *TaPIF3-2D*, *TaPIF4-5B/D*, and *TaPIF5-5A/B/D* were down-regulated (Figure 7B, Table S6), which may be due to the existence of LTR element in promoter region of *TaPIF1-1B*, *TaPIF2-2A/D*, *TaPIF3-2A*, *TaPIF5-5B/D*, *TaPIF6-5D* (Figure 4).”

“*TaPIF3-2A/B*, *TaPIF4-5A/B/D*, *TaPIF5-5A/B/D*, and *TaPIF6-5A/B/D* were down-regulated, while *TaPIF2-1B/D* were up-regulated in response to SA treatment, although only *TaPIF3-2A*, *TaPIF5-5D*, and *TaPIF6-5D* had SA-responsive elements in their promoters*.* The expression of all *TaPIFs* were *inhibited* by 6-BA treatment; *TaPIF1-1A/B*, *TaPIF2-1A/B/D* were down-regulated, while *TaPIF3-2A/B/D*, *TaPIF4-5A/B/D*, and *TaPIF5-5A/B/D* were up-regulated in response to ABA exposure compare to control, which were inseparable from the ABRE-element of their promoters (Figure 7C, Table S6). For GA and JA treatments, except that the expression level of *TaPIF5-5A/B/D* had no significance, the remaining *TaPIF* genes were down-regulated under GA treatments; *TaPIF2-1A/B/D*, *TaPIF4-5A/B/D*, and *TaPIF5-5A/B/D* had obvious response to exposure to JA (Figure 7C, Table S6).”

(7) Tests for statistical difference can be incorporated into the analyses, such as in the qRT-PCR results (Figure 8).

Response: According to reviewer's suggestion, we conducted statistical difference in the qRT-PCR, and the analytical method and significance of the data are described in the figure notes. See Figure 8 and Page lines 711-712.

“The asterisks indicate significant differences based on the results of the one-way ANOVA test (* *P* < 0.05; ** *P* < 0.01).”

Below are some minor issues of this manuscript:

(1) In Figure 3, the intron-exon-UTR results should be presented first (either as panel A or rearranged to the left of the figure). This is because this dataset is referred to in the manuscript text before the motifs.

Response: We appreciate the reviewer's suggestion. We have rearranged the intron- exon-UTR results to the left of the Figure 3.

(2) Lines 197-198: TaPIF2 also has high expression in “leaves, flag leaves, and young spikes”, but it was omitted from this sentence.

Response: According to the reviewer's suggestion, the omitted description has been added to Page 9, Lines 188-191.

“*TaPIF1s* had high expression in stem, flag leaves, and young spikes; *TaPIF2s* has high expression in leaves, flag leaves, and young spikes; *TaPIF3s*, *TaPIF4s*, and *TaPIF5s* were highly expressed in the stem, leaves, flag leaves, and young spikes; *TaPIF6s* were most highly expressed in leaves and flag leaves.”

(3) Lines 199-200: TaPIF6 was stated to be highly expressed in vegetative growth. However, its high expression is mostly limited to the roots; this alone should not constitute vegetative growth.

Response: The authors would like to thank the reviewer for their time and careful attention to detail. We have revised the description to make the conclusion more accurate, see Page 9, Line 199.

“ *TaPIF6s* were most highly expressed in leaves and flag leaves.”

Overall, we have made several modifications to the manuscript and done our best to thoroughly address all of the reviewers’ concerns. We again thank the editor and reviewers for their invaluable critique, comments, and suggestions, all of which have helped to improve our manuscript.

We kindly thank you again for your consideration!

Sincerely yours,

Dr. Tianqing Chen

Shaanxi Provincial Land Engineering Construction Group Co., Ltd. Xi'an, China.
