# Supplementary material for: Genome-wide identification and comprehensive analysis of the phytochrome-interacting factor (PIF) gene family in wheat
Source: PLoS One. 2024 Jan 5;19(1):e0296269. doi: 10.1371/journal.pone.0296269 (PMC10769075; doi:10.1371/journal.pone.0296269)
Supplement: Attachment — Submitted filename: Response to Reviewers.doc [file pone.0296269.s012.doc]

#### **Reply to the editors and reviewers**

#### Reference: PONE-D-23-27525

Title: Genome-wide identification and comprehensive analysis of the phytochrome-interacting factor (*PIF*) gene family in wheat

Authors: Hua Zhuang, Zhen Guo, Jian Wang, Tianqing Chen

Dear editors,

Thank you for your thorough review and patient guidance and offer our thanks to the reviewers for their thoughtful comments and suggestions regarding our study. We have carefully read and addressed reviewers’ suggestions in the manuscript and have done our best to address all concerns raised. Special attention has been given to rectifying details and made every effort to minimize errors in spelling and grammar, striving to enhance the overall quality of the paper. We are very grateful for the editorial and scientific guidance provided in this review process, which has greatly improved the quality of our study and will inform our research going forward. Once again, we appreciate your valuable feedback, and we look forward to any further guidance you may provide.

**Response to Reviewer #1**

There is a fundamental and serious error in the manuscript, namely that the number of the latest defined Arabidopsis PIFs is 8, not 7 (Lee and Choi 2017, jiang et al., 2022). Authors need to refer to the latest literature results, not those from 10 years ago (Leivar and Quail, 2011). All subsequent results and conclusions are therefore out of the question, and this error was pointed out last time.

Response: The authors appreciate the reviewer's suggestions. We understand the concerns about the accuracy of the reference sequence, fearing that the identification of PIF family members in wheat may be incomplete. In response to the reviewer's suggestions, we referenced the work of Lee and Choi (2017) and included the previously overlooked *Arabidopsis* AtPIF2 (AT2G46970) protein. We employed BLASTp to search for proteins in wheat with high similarity to AtPIF2, using a criterion of alignment length greater than 80 amino acids and similarity exceeding 50%. The identified proteins are TraesCS5A02G376500, TraesCS5B02G380200, TraesCS5D02G386500, TraesCS1A02G083000, TraesCS1B02G100400, and TraesCS1D02G084200, corresponding to our designated TaPIF5-5A, TaPIF5-5B, TaPIF5-5D, TaPIF1-1A, TaPIF1-1B, and TaPIF1-1D. These results did not alter the number of TaPIF family members identified in our previous characterization of wheat. Based on these findings, we incorporated AtPIF2 for multiple sequence alignment and phylogenetic analysis, subsequently revising Figure 1 and Figure S1.

“These include eight identified PIFs in tomato and apple [11, 12], respectively, and six identified PIFs in rice (*Oryza sativa*) and *Brachypodium distachyon* [13, 14], eight reported PIFs in Arabidopsis [11], potato [15], and maize (Zea mays) [16], respectively ”

**Response to Reviewer #2**

I greatly appreciate the authors’ efforts in revising the manuscript according to my previous suggestions. The authors have addressed most of my major concerns. The data and conclusions in this study are now generally convincing. However, due to some errors in the writing and figures, the manuscript is unfortunately not ready for publication.

Response: Thank you for your thoughtful review and constructive feedback. We appreciate your acknowledgment of our efforts in revising the manuscript and addressing your major concerns and glad to hear that the data and conclusions are generally convincing. We apologize for any remaining errors in writing and figures that may have hindered the publication readiness of the manuscript. We will carefully review and rectify these issues promptly to ensure the overall quality of the paper. Your insights are invaluable, and we are committed to delivering a polished and error-free manuscript. Once again, thank you for your time and feedback. We look forward to submitting the revised version for your consideration.

Below is a list of minor concerns that the authors should address:

(1) Line 92: There is no “TaPIF7-5D” after the reanalysis.

Response: The authors are very grateful for the reviewer's meticulous review, which helped identify our oversights. We have revised the corresponding descriptions to align them with the results of the reanalysis (Page 5, Line 90).

“TaPIF protein length ranged from 336 (TaPIF4-5A) to 516 (TaPIF5-5D) amino acids in length .”

(2) Line 102: The number “0.562” should be written as “-0.562”, otherwise “-0.95 to 0.562”will be interpreted as ranging from a negative to positive number.

Response: We appreciate the reviewer's suggestions. We have made the necessary corrections in the revised manuscript to avoid any misunderstandings (Page 5, Line 102).

“the hydrophilicity of PIF protein in *Arabidopsis* (-0.95 to -0.562) were generally higher than that in wheat.”

(3) Line 108: Instead of “ZmPIFs and BdPIFs”, did the authors mean “OsPIFs and ZmPIFs”? In figure 1A, BdPIFs distributed across all four groups.

Response: We appreciate the reviewer's suggestions, and we have revised the corresponding section accordingly (Page 6, Line 107).

“TaPIFs and BdPIFs were distributed among all four groups, ZmPIFs and OsPIFs were clusterd in group I, group III, and group IV.”

(4) Line 115: “Identified” should not itacilized.

Response: The authors appreciate the reviewer's attention to important details, and they have modified them to the correct format in the revised manuscript (Page 6, Line 114).

“TaPIF genes that were identified could be clustered into 6 homoeologous groups in wheat.”

(5) Line 121-122: Typo error, as “18 collinear pairs” was repeated.

Response: We appreciate the reviewer's suggestions, and repeated characters have been removed in the revised manuscript (Page 6, Line 121-122).

“These duplication events resulted in 23 collinear pairs of *TaPIFs* in the wheat genome, of which 18 collinear pairs were due to genome-wide duplication (WGD) (Figure 2A; Table S3) .”

(6) Line 122: “Genome-wide replication” is abbreviated wrongly.

Response: The authors appreciate the reviewer's suggestions, and corrections have been made in the revised manuscript to ensure consistency between the full term and its abbreviation (Page 6, Line 119).

“These gene replication events resulted in 23 collinear pairs of *TaPIFs* in the wheat genome, of which 18 collinear pairs were due to genome-wide duplication (WGD).”

(7) Line 127: Additional comma.

Response: We appreciate the reviewer's suggestions. We have already made the corrections.

“Notably, chromosomes 5A/B/D of wheat exhibited the highest homology with *O. sativa* and *B. distachyon*. *PIF* genes had been subject to strong purifying selection in rice and *B. distachyon* as their Ka/Ks ratios are less than 1 [43]”

(8) Line 127-128:“*PIF* genes had been subject to strong purifying selection in rice and *B. distachyon*”should be substantiated with data or by citing a reference. Similarly, the claim that“similar number of gene duplication events were found between *B. distachyon*, *O. sativa* and wheat, respectively”should be supported with evidence on the number of duplication events for each comparison.

Response: We appreciate the reviewer's suggestions. We have made modifications and additions to enhance the credibility of our descriptions.

“Notably, chromosomes 5A/B/D of wheat exhibited the highest homology with *O. sativa* and *B. distachyon*. *PIF* genes had been subject to strong purifying selection in rice and *B. distachyon* as their Ka/Ks ratios are less than 1 [43], and the similar number of gene duplication events was found in wheat/*O. sativa* (24) and wheat/*B. distachyon* (25), respectively.”

(9) Line 145: In Figure S2, motif 2 and 5 do not match with the known sequences of bHLH and APB domains, respectively. This part should be revised after careful analysis of the results. By comparing with published consensus sequences, motif 1 appears to be the bHLH domain while motif 2 matches closely with the APB domain. The authors can demonstrate this by comparing the identified motif sequences in figure S2 with published bHLH and APB domain sequences (via sequence alignment).

Response: We appreciate the reviewer's suggestions. After careful comparison and verification, we have made modifications in the revised manuscript to ensure the accuracy and reliability of our description (Page 7, Lines 146-147).

“All of the TaPIFs contained the same arrangement of motif 1 and motif 2, representing bHLH and APB domains, the features of PIF proteins, respectively.”

(10) The authors should note that for AtPIFs, the APB domain tends to be in the N-terminus while the bHLH domain is found in the C-terminus. This is not conserved for all Arabidopsis bHLHs. Since, this pattern (APB domain in N-terminus and bHLH domain in C-terminus) is also observed in all TaPIFs, it strongly supports the claim that the identified proteins are functionally similar to Arabidopsis PIFs. The authors can consider referring to “The Basic Helix–Loop–Helix Transcription Factor Family in Plants: A Genome-Wide Study of Protein Structure and Functional Diversity” by Heim et al. (2003) (Molecular Biology and Evolution).

Response: The authors are deeply grateful for the valuable suggestions provided by the reviewer, which have been instrumental in enhancing the quality of our work. We have added further description in this section in the discussion to make our analysis more comprehensive (Page 13, Lines 284-287).

“Additionally, we have observed that the APB domain and bHLH domain tend to be located at the N-terminus and C-terminus in AtPIF proteins, respectively (Figure S2). This similar arrangement pattern is also observed in all TaPIFs proteins, which strongly supports the claim that the identified TaPIF proteins were functionally similar to AtPIF proteins [49].”

(11) Line 251-253:“Transactivation assays were performed in yeast with TaPIF2 and TaPIF4 .Each gene was cloned from the wheat cultivar ‘Chinese Spring’”. Which TaPIF2 gene was cloned – TaPIF2-1A, TaPIF2-1B, or TaPIF2-1D? Likewise, which TaPIF4 gene was cloned?

Response: The authors appreciate the reviewer's suggestions. We cloned *TaPIF2-1D* and *TaPIF4-5A* as a representative to verify their transcriptional activation and subcellular localization due to the high similarity among homologous genes as mentioned earlier (see Page 5, lines 88-90). In response to the reviewer's comments, we have supplemented this information in the revised manuscript (see Page 12, lines 250-254; Lines 258-260) and made corresponding modifications in Figure 9.

“Due to the high similarity among homologous genes, One copy of homologous was selected for experimental validation. *TaPIF2-1B* and *TaPIF4-5B* were cloned from the wheat cultivar ‘Chinese Spring’, inserted into the vector pGBKT7, and transformed into yeast. TaPIF2-1B and TaPIF4-5B promoted increased yeast growth on SD/-Trp/-His/-Ade medium compared to yeast transformed with the empty vector negative control .”

“we developed transient expression vectors to express plasmids encoding TaPIF-GFP fusion proteins (TaPIF2-1B-GFP and TaPIF4-5B-GFP) in wheat protoplasts.”

(12) Line 266-268: Please revise this sentence. While the meaning is generally understood, the sentence is too long and has too many commas, making it unclear.

Response: We appreciate the reviewer's suggestions. We have revised the sentence to make it clear and concise (Page 12, Lines 266-269).

“In this study, we identified 18 PIF genes in wheat, categorized into 6 homeologous groups (Table 1), which is a greater number of PIF genes than in the Arabidopsis, rice, maize, or B. distachyon genomes (Table S1). This increase is likely due to wheat being a heterohexaploid with three subgenomes.”

(13) Line 269: “whole genome replication” was abbreviated wrongly.

Response: The authors appreciate the reviewer's suggestions, and corrections have been made in the revised manuscript (Page 12, Lines 269-270)

“Polyploid events or whole genome duplication (WGD) and amplification events play an important role in the genome evolution of plant species .”

(14) Lines 272-273: “Here, collinearity analysis of wheat, O. sativa, and B. distachyon revealed 21, seven, 24, and 25 collinearity PIF gene pairs, respectively”. There are three plant species and four collinearity PIF gene pairs. Please clarify this.

Response: We appreciate the reviewer's suggestions. We have corrected this error in the revised manuscript (Page 13, Lines 272-274).

“Here, collinearity analysis of wheat/wheat, wheat/*O. sativa*, and wheat/*B. distachyon* revealed 21, 24, and 25 collinearity PIF gene pairs, respectively.”

(15) Lines 334-336: The evidence shown in Figure 6B did not include all three genes for TaPIF2 nor TaPIF4. It is therefore erroneous to claim that the subcellular localization assay confirmed the location of all three TaPIF2 and TaPIF4.

Response: We appreciate the reviewer's suggestions. As suggested by the reviewer in point 11 (see Page 5, lines 89-91; Page 12, lines 255-258, Line 264 ), we have made the necessary corrections in the main text (see Page 16, lines 345-368) and Figure 9 .

“In vitro transcriptional activity assays with TaPIF2-1D and TaPIF4-5A, provided empirical evidence that at least some TaPIFs serve as transcriptional activators (Figure 9A). Furthermore, subcellular localization assays confirmed that the three TaPIF2-1D and TaPIF4-5A were localized to the nucleus as predicted (Figure 9B) ”.

(16) Figure legends for Figure 2B: Please state what the orange and green bars represent.

Response: The authors are grateful for the reviewer's suggestions. We have incorporated the corresponding descriptions in the figure legends for better reader comprehension (Page 30, lines 679-681).

“The orange bars represent chromosomes in wheat, while the green bars represent chromosomes in rice and *B. distachyon*. The text on the sides of the bars indicates the chromosome numbers.”

(17) Figure 7: TaPIFs included TaPIF7 and TaPIF8, which were not part of the list of TaPIFs after the re-analysis. The manuscript text does not correspond to the figure.

Response: We appreciate the reviewer's suggestions. The authors apologize for the oversight, and the accurate names have been rectified in Figure 8.

(18) Figure legends for Figure 7: Please state what the color bars represent. Are they z-scores or relative expression?

Response: We appreciate the reviewer's suggestions. We have added explanatory notes in the figure legend of Figure 6 and 7 to clarify the meaning represented by the color bars (Page 28, lines 708-709; Page 30, lines 719-721).

“The color bars represent the level of gene expression, with blue indicating low expression and red indicating high expression.”

(19) Figure 8: The time points for TaPIF2 (both PEG and ABA treatment) are incorrect. The time points are “3, 6, 12, 24, 12, 24h”.

Response: The authors are sincerely grateful to the reviewer for identifying this crucial error. We have made the necessary corrections in Figure 8.

(20) Table S7: Last primer is labelled as TaPIF5_GFP. Is this a typo error and that it should be TaPIF4?

Response: We appreciate the reviewer for pointing out this error. We have rectified it in Table S7.

(21) Generally, the writing can be further revised to improve sentence structure. Some sentences were too long with multiple punctuation, which makes it difficult to read. There are also multiple labelling and typo errors throughout the manuscript (there are additional typo errors that are not specifically mentioned in this review). The authors should thoroughly check the writing before resubmission.

Response: Thank you for your thoughtful review and constructive feedback on our manuscript. We appreciate your insights into the need for further improvement in sentence structure. We acknowledge your observations regarding lengthy sentences with multiple punctuations and the presence of labeling and typo errors throughout the manuscript. We have take your comments to heart and conduct a thorough review of the writing to address the mentioned issues and additionally submitted the revised manuscript to a professional editing service to correct and polish the English. Your feedback is invaluable in enhancing the overall quality of our paper.

Overall, we have made several modifications to the manuscript and done our best to thoroughly address all of the reviewers’ concerns. We again thank the editor and reviewers for their invaluable critique, comments, and suggestions, all of which have helped to improve our manuscript.

We kindly thank you again for your consideration!

Sincerely yours,

Dr. Tianqing Chen

Shaanxi Provincial Land Engineering Construction Group Co., Ltd. Xi'an, China.
